# Arc Dynamics and Material Erosion of Pantograph–Catenary Contacts Under a Rotating Permanent-Magnet Field

**DOI:** 10.3390/ma19214563

**Published:** 2026-10-29

**Authors:** Bingquan Li, Huajun Dong, Ningning Wei, Ran Ji, Lei Huo

**Affiliations:** 1 Department of Railway Rolling Stock, Liaoning Railway Vocational and Technical College, Jinzhou 121000, China; libingquan@lntdxy.edu.cn (B.L.);; 2 School of Mechanical Engineering, Dalian Jiaotong University, Dalian 116028, China; 3 School of Energy and Mechanical Engineering, Dezhou University, Dezhou 253023, China; 4 School of Materials Science and Engineering, Dalian University of Technology, Dalian 116024, China; lh@mail.dlut.edu.cn

**Keywords:** rotating permanent magnet, pantograph–catenary arc, arc-root migration, current-collection characteristics, arc erosion, SEM/EDS

## Abstract

To mitigate localized erosion of pantograph–catenary system (PCS) contacts caused by random migration of arcs during contact loss, a periodic transverse magnetic field generated by a rotating permanent magnet was proposed to regulate arc motion. A synchronized high-speed imaging and electrical-signal acquisition platform was established to compare arc dynamics, current-collection characteristics, and carbon-electrode erosion under no field, a static field, and periodic fields of 5, 10, and 30 Hz at 120 V DC and 25 A, while SEM/EDS characterized surface morphology and elemental composition. The static field shortened the arcing duration and continuously stretched the arc column toward one side, but produced directional surface grooves. Under periodic fields, the arc column alternately deflected and contracted. Among rotating-field cases, 10 Hz yielded a normalized RMS current ratio of 72.923% and the lowest current fluctuation coefficient of 13.509%, whereas 30 Hz showed no further improvement in electrical or erosion performance. These results indicate that erosion mitigation is associated with both arcing energy and arc-root attachment-site renewal, and that the half-cycle at 10 Hz provides a favorable balance among arc migration, current-collection stability, and thermal-load dispersion, providing experimental guidance for selecting magnetic-drive parameters for PCS arcs.

## 1. Introduction

The traction power supply system is a critical infrastructure of urban rail transit, while the PCS forms the dynamic current-collection interface between the train and the traction network. Unlike electrified railways, which commonly employ a 27.5 kV alternating-current (AC) traction power supply, most urban rail systems use a 1500 V direct-current (DC) rigid overhead catenary to better accommodate equipment installation within confined tunnel spaces [1,2]. However, because rigid overhead catenary systems lack conventional tension-compensation and elastic components, pantograph–catenary contact is more sensitive to operating speed and structural irregularities, which can induce contact-force fluctuations and even transient contact loss [3]. When the electric-field strength across the separation gap exceeds the dielectric withstand strength of air, collision ionization occurs and a conductive plasma channel is formed, resulting in a pantograph–catenary arc [4]. The intense thermal flux generated by the arc can cause ablation, melting, and material transfer at the contact wire and pantograph strip, thereby further degrading the sliding electrical contact condition and current-collection quality of the PCS [5]. Therefore, developing effective approaches for actively regulating pantograph–catenary arcs and clarifying the associated dynamic response and material-erosion mechanisms are essential for improving the current-collection reliability and service life of the PCS.

The wear behavior of pantograph–catenary contact pairs is jointly influenced by environmental conditions, material properties, and electrical loading [6,7]. In conventional mechanical friction systems, external magnetic fields have been introduced to improve the tribological performance of contact pairs, with previous studies reporting reduced metallic wear and increased surface hardness under magnetic-field conditions [8,9]. For current-carrying friction systems, Ref. [10] developed an arc-ablation model for the PCS and showed numerically that the introduction of a magnetic field can redistribute current density, reduce current-collection losses, and decrease the probability of arc occurrence. Recent studies have further demonstrated the important role of magnetic fields in regulating arc dynamics. Qian et al. [11] developed a chain model of PCS arcs incorporating the mass of current elements and divided arc evolution into three stages, showing that electromagnetic forces can dominate the initial and rapid-stretching stages and thereby govern magnetically driven arc migration. Yu et al. [12] investigated the arc temperature field and magnetic-field distribution using a multiphysics finite-element method and found that the magnetic field can alter both the arc-energy transfer path and the spatial distribution of thermal diffusion. Similarly, Li et al. [13] employed a magnetohydrodynamic (MHD) model and numerical simulations of vacuum arcs and showed that an externally applied transverse magnetic field can modify the deflection trajectory, plasma-energy distribution, and electrode-surface heating of high-current vacuum arcs. Scanning electron microscopy and energy-dispersive X-ray spectroscopy (SEM/EDS) studies have also shown that arc erosion and thermal stress jointly produce complex surface-ablation features, particularly in copper-based conductors widely used in catenary systems [14], while the application of an external magnetic field can reduce copper transfer and promote a transition of the contact materials from severe to mild wear [15]. Although previous studies have demonstrated the feasibility of magnetic-field-assisted arc regulation, the relationship between the field-reversal timescale, arc-root migration, electrical conduction, and cumulative surface erosion remains insufficiently characterized within a unified pantograph–catenary contact-loss experiment. Compared with coil-excited electromagnetic modules, a mechanically rotated permanent magnet provides periodic magnetic-field reversal without coil excitation or the associated winding losses, although motor power consumption and rotation-induced disturbances remain relevant engineering considerations.

This study investigates the effects of a transverse magnetic field on the arc dynamics, electrical behavior, and material erosion of the PCS. A rotating-permanent-magnet module was integrated into an existing reciprocating current-carrying arc-generation platform, with the magnet position and nominal field setting kept unchanged while the rotation frequency was varied as the primary control parameter. Test conditions included no magnetic field, a static magnetic field, and periodic magnetic fields at different rotation frequencies. Synchronized high-speed imaging and voltage–current acquisition were used to characterize arc-column morphology, arc-root migration, and electrical and fluctuation-related parameters, while carbon-strip damage was quantified at a consistent macroscopic scale and further evaluated by SEM/EDS to assess the material response. The results clarify the influence of magnetic-field frequency on arc dynamics and current-collection quality, showing that 10 Hz produced lower current fluctuations, higher normalized RMS arcing-current ratio, and more repeatable arc-root migration among the rotating-field conditions tested. These findings provide an experimental basis for mitigating localized erosion of carbon strips and optimizing magnetic-drive parameters for PCS arcs.

## 2. Experimental Methodology

### 2.1. Experimental Materials

**Figure 1:**
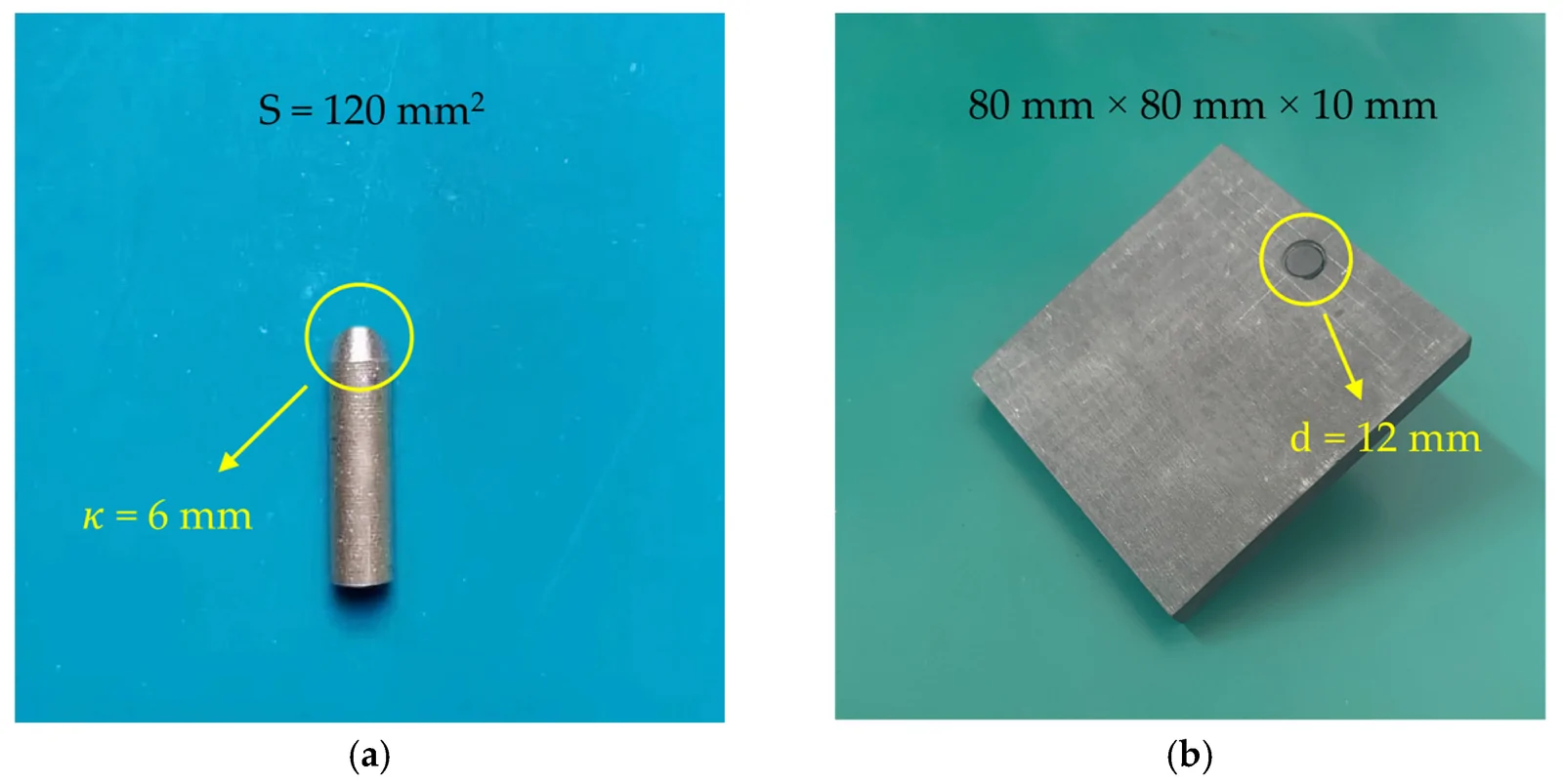
The contact-pair specimens (adapted from [17]). (**a**) Pure copper rod (Cu-CATH-2); (**b**) pure carbon plate.

Contact wires in urban rail systems commonly employ CTA120 copper–silver alloy, in which Cu accounts for approximately 99.74 wt.% and Ag for approximately 0.10 wt.% [16]. Accordingly, a high-purity copper rod (Cu-CATH-2) was used on the contact-wire side in this study, and its contact end was locally machined with reference to the profile of a CTA120 contact wire, resulting in a cross-sectional area *S* of approximately 120 mm^2^ and an end curvature radius *κ* of approximately 6 mm. Owing to its excellent heat resistance and high sublimation temperature, a carbon-based material with a carbon content of no less than 98% was used on the slider side and machined into rectangular specimens measuring 80 mm × 80 mm × 10 mm to accommodate the clamping fixture. To ensure reliable fixation, a threaded hole with a diameter *d* of 12 mm was machined at a prescribed position in the upper-middle region of each carbon specimen, into which a 25 mm long carbon rod of the same material was inserted to provide both mechanical fixation and electrical connection. To ensure stable and repeatable contact conditions between the contact pair, all specimens were produced from the same material batches and machined according to identical processing specifications. The machined specimens used in the experiments are shown in Figure 1.

Material composition and thermophysical properties are important factors governing resistance to arc erosion [18]. Table 1 summarizes the available physical and mechanical properties of the selected contact-pair materials.

### 2.2. Experimental Platform

**Figure 2:**
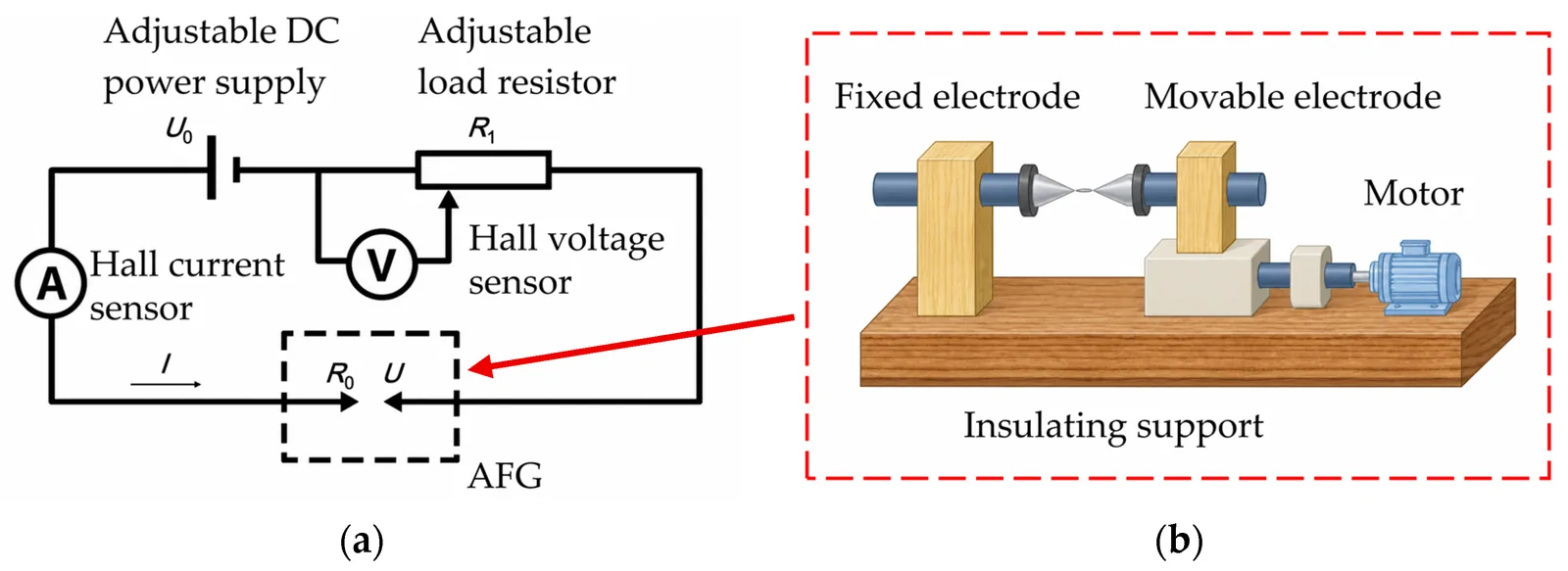
Electrical connections and structural configuration of the AFG. (**a**) The electrical connection principle; (**b**) the general configuration of the AFG. Here, *I* denotes the circuit current, *R*_0_ the equivalent resistance of the AFG contact/discharge region, and *U* the interelectrode voltage.

In pantograph–catenary systems for rail transit, the contact wire is typically installed with a prescribed lateral stagger along the track, causing the contact point to migrate continuously across the width of the pantograph strip and thereby promoting more uniform strip wear [19], which results in a characteristic zigzag sliding trajectory. This arrangement means that the actual contact process involves both relative sliding in the train-running direction and lateral displacement of the contact wire. However, most existing arc studies rely on an arc-fault generator (AFG), which consists of a movable electrode and a fixed electrode and generates arcs through single-path electrode separation [20], making it difficult to reproduce the coupled motion characteristics of actual pantograph–catenary contact pairs. Figure 2 shows the electrical connection and structural configuration of the AFG.

In this study, the motion mechanism of the AFG was modified to provide the movable electrode with two independently speed-controlled degrees of freedom, namely lateral sliding and vertical separation. Based on the existing current-carrying separation test platform [17], a synchronized multi-source experimental platform integrating magnetic-field control, electrical measurements, and high-speed imaging was established. The platform mainly consists of a DC power-supply circuit, a two-degree-of-freedom electrode motion mechanism, a multichannel data acquisition (DAQ) system, an environmental boundary-control unit, a high-speed imaging system, and a magnetic-field regulation module.

The power supply was provided by a programmable DC source (IT6018D-1500-40, ITECH, Nanjing, China) with a maximum output voltage of 1500 V and a full-scale accuracy error below 0.02%. The source was configured in constant-voltage (CV) priority mode, with the output-voltage setpoint maintained at 120 V. The external series load resistance was adjusted to establish the prescribed initial current, while the current was allowed to vary with the evolving discharge conditions during arcing.

To reduce environmental interference during the magnetic-field experiments, the experimental platform was equipped with an enclosed arc-generation chamber whose walls were covered with low-transmittance polyethylene terephthalate (PET) thermal-insulation film to reduce interference from ambient light. Two orthogonally stacked nonmagnetic dual-rail linear stages, each driven by an independent stepper motor, were installed inside the chamber. The specimens were mounted on the stages using insulating epoxy-resin fixtures, with the copper specimen fixed to the lower stage and the carbon specimen mounted on the upper stage in a face-to-face configuration. The two motion axes were independently controlled to perform lateral sliding and vertical separation, allowing the contact pair to transition gradually from stable current-carrying contact to arc discharge after contact loss. Lateral displacement was used to simulate the relative pantograph–catenary sliding induced by the contact-wire stagger, whereas vertical displacement controlled the increase in the separation gap. Four limit switches were installed at the edges of the stages to constrain the initial positions and effective travel ranges, thereby improving repeatability among different test conditions. To meet the requirements of the externally applied magnetic-field experiments, all moving and clamping components were made of nonmagnetic materials to minimize disturbance of the magnetic field in the arc region.

During arc discharge, the arc voltage and circuit current were measured using a Hall-effect voltage sensor (CHV-50P, SENSOR Electronics, Beijing, China) and a Hall-effect current sensor (CHB50-SF, SENSOR Electronics, Beijing, China), with measurement ranges of 1000 V and 50 A, respectively, and measurement errors below 0.8%. The voltage measurement terminals were connected to the bases of the two electrodes, while the current sensor measured the cathode-branch current close to the test chamber to reduce the influence of line voltage drop and parasitic impedance on the electrical measurements. A thin-film pressure sensor was used to apply and verify the initial normal contact load. During the experiments, the environmental conditions inside the chamber were maintained consistently and monitored using a temperature–humidity sensor (SHT31-ARP-B, SENSIRION, Stäfa, Switzerland) and an oxygen concentration sensor (LFO2-A1, Alphasense, Great Notley, England). After electrical isolation and signal conditioning, all sensor signals were acquired synchronously by a multichannel DAQ system (USB-3123, Smacq, Beijing, China) with a maximum hardware sampling rate of 1 MSa/s. For the electrical analysis, the voltage and current signals were recorded at a sampling rate of 1 kSa/s.

The high-speed camera was focused on the arc-generation region within the contact-pair gap to continuously record arc-column deformation and arc-root migration. A polarizing filter assembly was mounted in front of the camera lens to reduce image saturation caused by intense arc radiation and reflections from the electrode surfaces. The recording frame rate was set to 1250 frames per second (fps) with an image resolution of 720 × 720 pixels (px). In addition, when the DAQ system was activated, a low-voltage trigger signal was simultaneously sent to the high-speed camera to establish a common time base between the electrical waveforms and high-speed images, providing synchronized data for subsequent analyses of arc-root migration and electrical response.

The magnetic-field regulation module mainly consists of a permanent magnet, a nonmagnetic coupling, a servo motor, a shaft encoder, and a magnetic-field measurement unit. A high-temperature-resistant samarium–cobalt magnet (Sm_2_Co_17_) was used as the permanent magnet and was magnetized normal to its broad face, with the magnetization direction perpendicular to the motor shaft. The magnet was mounted on the motor shaft through a nonmagnetic coupling and an L-shaped aluminum-alloy bracket, and the maximum motor speed was 5000 r/min. The permanent-magnet assembly was positioned on one side of the arc region, and the loading position was determined by adjusting the distance between the magnet and the contact pair so that the rotational axis maintained a defined and repeatable spatial relationship with the center of the expected arc region. As the magnet rotated about its axis, the transverse magnetic-field vector in the arc region varied periodically, thereby imposing a magnetic driving force of alternating direction on the current-carrying arc column. Before the formal experiments, a preliminary multipoint Hall-probe survey was conducted over the expected arc region using a Hall gaussmeter (Model 475 DSP, Lake Shore Cryotronics, Inc., Westerville, OH, USA) equipped with a transverse Hall probe (HMMT-6J04-VR, Lake Shore Cryotronics, Inc., Westerville, OH, USA). This survey was used to guide magnet positioning and establish the nominal correspondence between the magnetic field at the arc-region center and an external reference position. During the experiments, the Hall probe was placed at this reference position with its sensitive axis aligned parallel to the target transverse magnetic-field component to verify the applied magnetic-field condition. Because the rotating components could introduce local airflow disturbances and mechanical vibrations, the magnet was maintained at a fixed distance from the arc region, and no additional blades or flow-guiding structures were installed. These measures avoided intentional aerodynamic forcing but could not completely exclude disturbances induced by mechanical rotation. 

**Figure 3:**
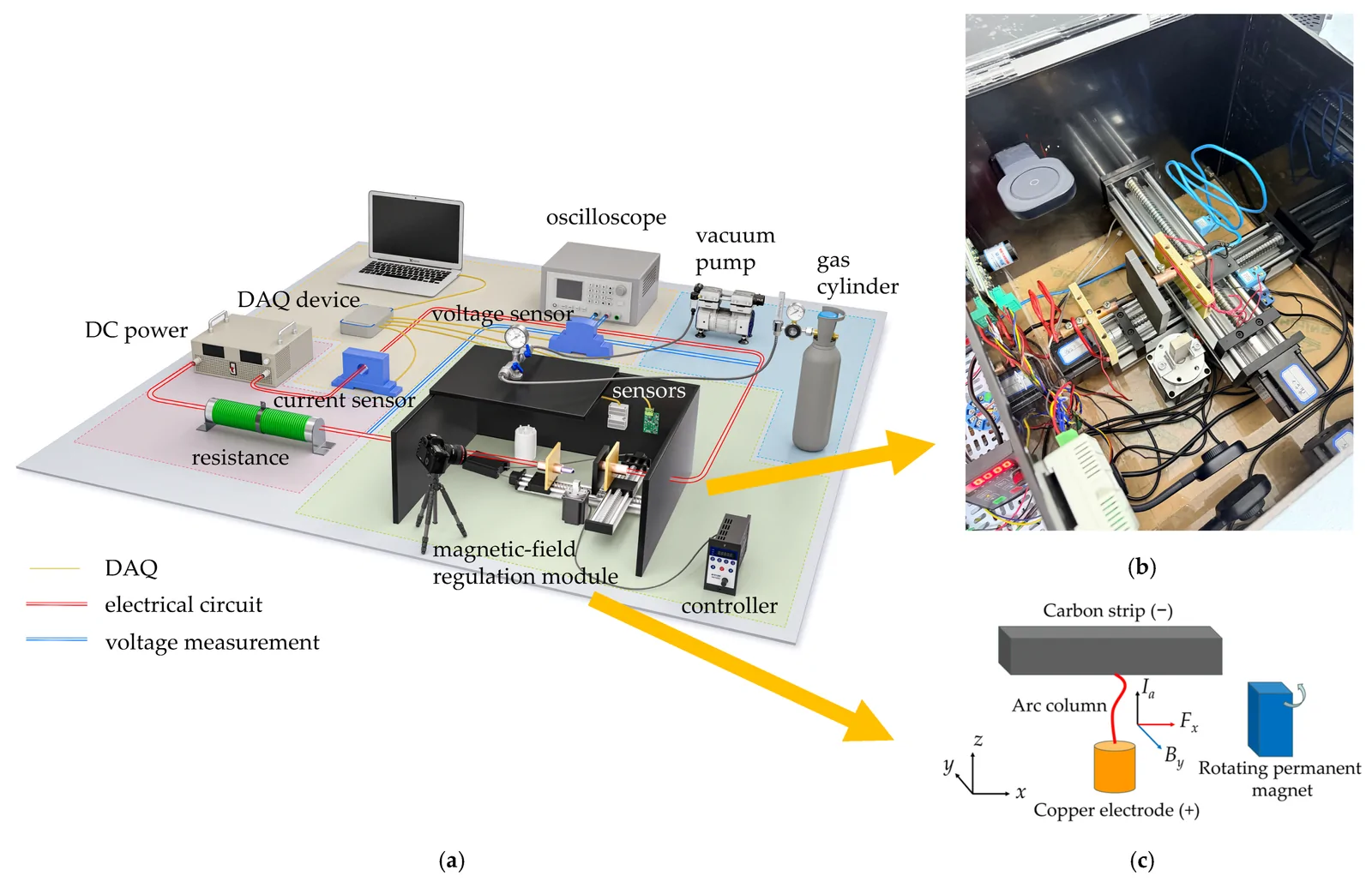
Experimental platform and spatial relationship among the electrodes, arc, and magnetic field. (**a**) Overall configuration and functional layout of the experimental platform; (**b**) arc generation chamber; (**c**) schematic illustration of the electrode arrangement, arc-current direction, transverse magnetic field, and resulting electromagnetic force. The arrows in (**c**) represent one phase of the rotating-field cycle. The auxiliary gas-handling components in (**a**) were not operated in the present experiments. In Figure 3a, the pink, beige, green, and blue shaded regions represent the power-supply and load circuit, data-acquisition and monitoring system, arc-generation and magnetic-field regulation module, and auxiliary gas-handling system, respectively.

The experimental platform also incorporates a vacuum pump and a gas cylinder as part of its auxiliary gas-handling system. However, these components were not used in the present magnetic-field experiments. All tests were conducted in ambient air without chamber evacuation or external gas injection. Figure 3 shows the experimental platform and the spatial arrangement of the electrodes, arc, and applied magnetic field.

### 2.3. Experimental Conditions

To isolate the coupled effects of environmental conditions, electrical loading, and contact-pair motion on magnetic-field regulation, all experiments were conducted under identical reference conditions. The temperature inside the test chamber was maintained at 23.0 ± 0.3 °C, the relative humidity at 50 ± 2% RH, and the total pressure at 101.325 ± 0.5 kPa, which were adopted as reference boundary conditions for the comparative experiments [21]. The copper specimen was connected to the positive terminal of the DC power supply and served as the anode, while the carbon specimen was connected to the negative terminal and served as the cathode. Before energization, an initial normal load of 50 N was applied and the initial contact resistance was confirmed to be *R* ≤ 10 mΩ. Based on the stable arcing conditions established in the preliminary platform tests, the DC source voltage was set to 120 V, and the series load resistance was adjusted to establish an initial contact-conduction current of approximately 25 A. The 120 V supply setting and approximately 25 A initial contact-conduction current represent controlled laboratory conditions rather than full-scale traction operation. Therefore, these electrical conditions were primarily intended for comparing arc responses under different magnetic-field configurations, and the quantitative findings should not be directly extrapolated to higher-voltage or higher-current conditions. Both the lateral sliding and vertical separation velocities were set to 10 mm/s, while identical initial positions and single-path motion were maintained using four limit switches installed on the linear stages.

A right-handed coordinate system was adopted, with the *x*-axis along the lateral sliding direction, the *z*-axis along the vertical electrode-separation direction and approximately parallel to the main arc-current path, and the *y*-axis normal to the imaging plane. The transverse magnetic-field component *B_y_* therefore produces the dominant magnetic driving force along the *x*-axis. The background magnetic field was determined with the permanent magnet removed and subtracted from the measured field under magnetic loading. Under uniform rotation, the transverse magnetic-field component at the center of the arc region can be approximated by its fundamental harmonic as follows:

*B_y_*(*t*)≈*B*_0_ + *B_p_*cos(2*πf_B_t* + *φ_B_*), (1)

where *f_B_* denotes the magnetic-field variation frequency, *φ_B_* is the initial phase, *B*_0_ represents the DC component arising from the background magnetic field and installation offset, and *B_p_* denotes the effective transverse magnetic-field amplitude. Because of the finite dimensions and single-sided arrangement of the permanent magnet, Equation (1) therefore provides an approximate fundamental-harmonic representation, while Equation (2) defines the half-range amplitude and midpoint offset that can be estimated from the extrema of a measured Hall waveform.

(2) $$B_{p}=\frac{B_{\max}-B_{\min}}{2},\;\;B_{0}=\frac{B_{\max}+B_{\min}}{2},$$

For a rotating permanent magnet with *p* pole pairs, the fundamental frequency *f_B_* of the transverse magnetic-field component is related to the mechanical rotational speed *n* (r/min) by:

(3) $$f_{B}=p\frac{n}{60},$$

A single-pole-pair permanent magnet *p* = 1 was used in this study. The distance between the magnet and the contact pair was adjusted using Hall-probe measurements to establish a nominal transverse magnetic-field amplitude at the arc-region center to *B_p_*_,*c*_ = 1.0 mT (10 G). The magnet position was then kept unchanged across all rotating-field conditions to maintain the same nominal spatial field configuration. The magnetic-field frequency was checked using the Hall signal at the reference position and compared with the value calculated from the encoder-measured rotational speed. To characterize the spatial variation in transverse magnetic-field amplitude relative to the arc-region center, the maximum relative deviation can be defined as:

(4) $$\delta_{B}=\underset{i}{\max}\left|\frac{B_{p,i}-B_{p,c}}{B_{p,c}}\right|\times 100\%,$$

where *B_p_*_,*i*_ denotes the transverse magnetic-field amplitude at the *i*th calibration point within the arc region, and *δ_B_* represents the maximum amplitude deviation of the calibration points relative to the arc-region center. The nominal field values in Table 2 refer to the arc-region center and do not imply a spatially uniform or perfectly sinusoidal magnetic field. The magnetic force acting on the current-carrying arc column depends on the local current direction and magnetic-flux density. For an infinitesimal line element d***l*** along the current-carrying arc column, the magnetic force exerted by the applied field is given by:

d***F****_L_* = *I_a_* d***l*** × ***B***, (5)

*I_a_* denotes the instantaneous arc current, d***l*** is oriented along the conventional current direction, and ***B*** represents the externally applied magnetic flux density at the corresponding line element. When the transverse magnetic-field component reverses direction, the lateral magnetic force changes direction accordingly and influences arc-root attachment through the resulting displacement of the arc column. Previous thermal-plasma experiments have observed periodic arc migration driven by low-frequency transverse magnetic fields, with the migration amplitude varying across different frequency ranges [22]. Five test conditions were therefore established, as listed in Table 2, in which the no-field and static-field cases served as the baseline and unidirectional-driving reference conditions, respectively, while the rotating-field cases were set to 5, 10, and 30 Hz, corresponding to 300, 600, and 1800 r/min and nominal half-periods of 100, 50, and 16.7 ms, respectively, to compare the arc response as the field-reversal time decreased.

A newly machined copper electrode from the same material batch was used for each magnetic-field condition, while the same electrode was retained for the three repeated runs within that condition. Before each test, the copper and carbon contact surfaces were lightly polished using 1000-grit abrasive paper, cleaned with ethanol to remove surface debris, and allowed to dry. Between repeated runs, the copper-electrode tip was additionally reconditioned to remove visible surface irregularities and approximately restore its original rounded profile. The electrode position, initial normal load, and contact resistance were then checked to ensure consistent initial contact conditions. The target magnetic-field condition was then established. For M1, the permanent magnet was withdrawn until the reference-probe reading returned to the background-field range. For M2, the magnet was fixed at the angular position corresponding to a transverse magnetic-field component of *B_y_* = +1.0 mT at the arc-zone center. For M3–M5, the magnet position and nominal field setting were kept unchanged, while the motor was operated at the prescribed rotational speed. After the magnetic-field reading at the reference position stabilized, the field condition was maintained and the DC circuit was energized to initiate the lateral-sliding and vertical-separation sequence. The DAQ system and high-speed camera synchronously recorded the voltage, current, and arc images. The power supply was switched off immediately after natural arc extinction, after which the data were saved and the carbon-strip surface morphology was documented. Each magnetic-field condition was tested in three repeated runs, with an interval of at least 30 min between consecutive runs to allow sufficient cooling before subsequent testing. 

After arcing, the arc-eroded regions of the carbon specimens were examined using a Merlin Compact field-emission scanning electron microscope (FE-SEM, Carl Zeiss, Germany), and the elemental composition of representative regions was analyzed by EDS. SEM observations were performed at an accelerating voltage of 5 kV using the SE2 detector at magnifications of 200×, 500×, 1000×, and 3000×. EDS analysis was conducted at an accelerating voltage of 15 kV with an effective acquisition time of approximately 60 s. For each test condition, EDS area analysis was performed on one representative arc-eroded region from one carbon specimen, and the elemental composition was reported as instrument-normalized mass fractions. Accordingly, the EDS results were used for descriptive comparison, without statistical evaluation of spatial or specimen-to-specimen variability.

The quantitative arc-root, electrical, and macroscopic erosion parameters were reported as mean ± standard deviation from three repeated runs. For the three rotating-field conditions (5, 10, and 30 Hz), exploratory pairwise comparisons of the macroscopic erosion parameters were performed using two-sided Welch’s *t*-tests, with Holm correction applied across the nine comparisons. Statistical significance was assessed at *p* < 0.05.

## 3. Results and Discussion

### 3.1. Arc Morphology Evolution

**Figure 4:**
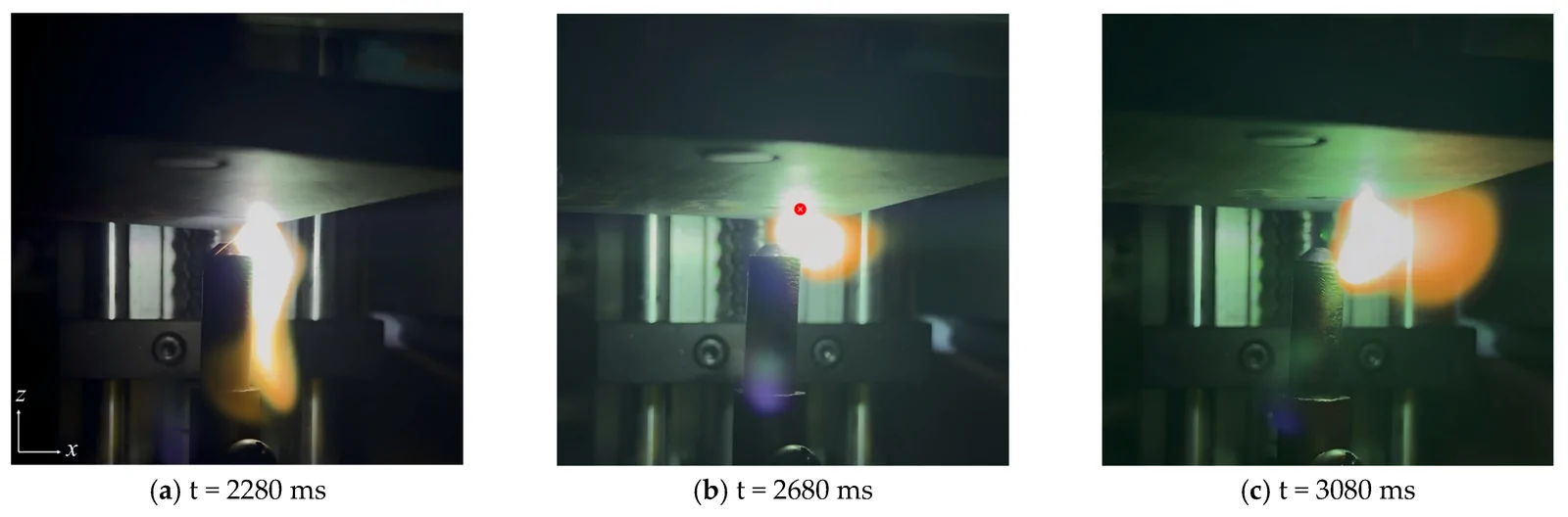

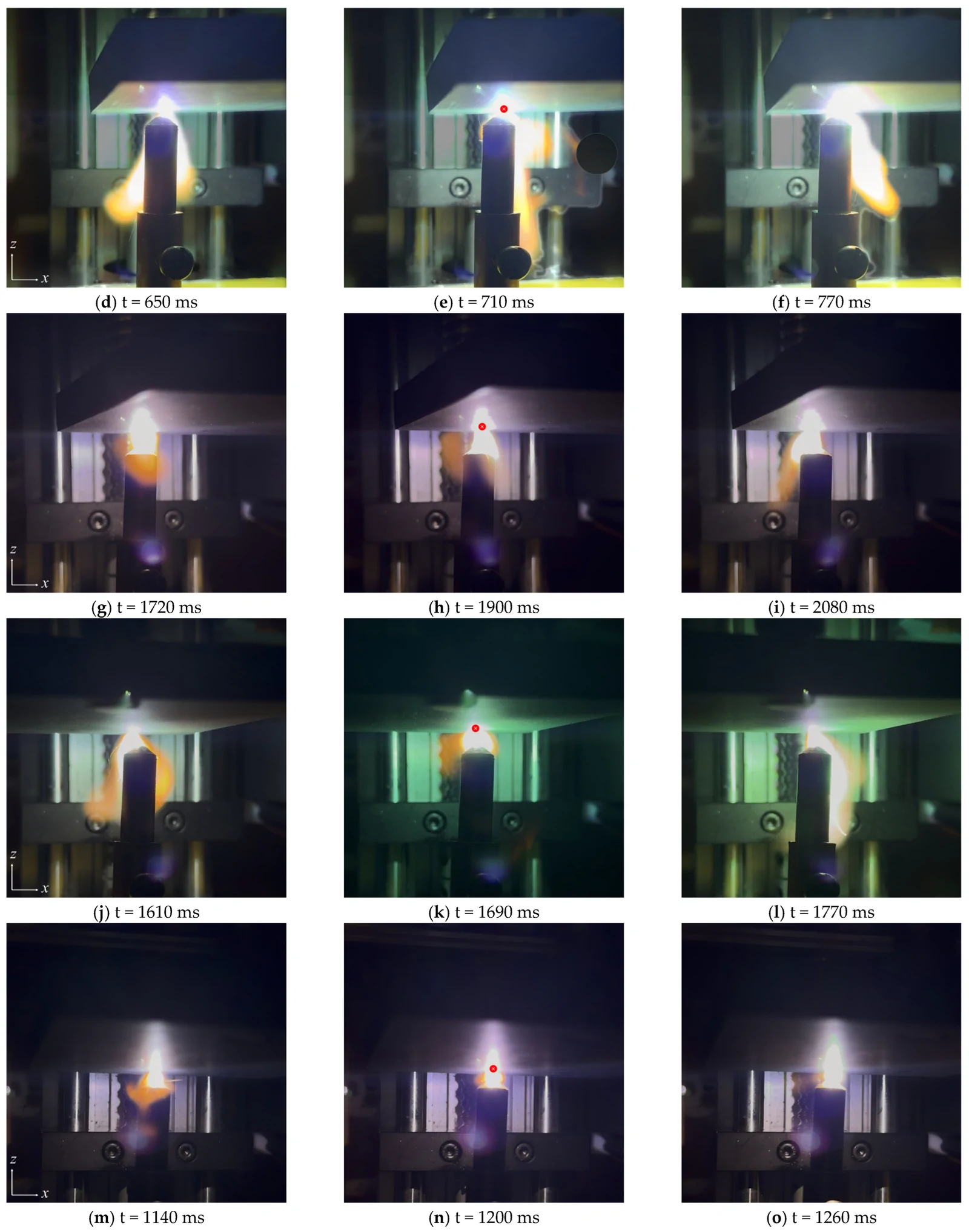
Typical arc morphologies under different magnetic-field conditions. (**a**–**c**) The no-field condition; (**d**–**f**) the 0 Hz condition; (**g**–**i**) the 5 Hz condition; (**j**–**l**) the 10 Hz condition; (**m**–**o**) the 30 Hz condition. Red crossed circles mark the optically estimated cathode arc-root positions in selected frames.

Arc-column deformation, arc-root detachment, and reattachment all require finite response times. Therefore, the half-period of the rotating magnetic field may determine whether the arc can follow the reversal of the external magnetic-force direction and complete a distinguishable migration process. Figure 4 shows representative arcing frames extracted from the contact-loss arc image sequences under different magnetic-field conditions.

The morphologies shown in Figure 4 do not represent five isolated phenomena, but rather a continuous evolution from unconstrained drift through sustained unidirectional forcing to periodically reversing magnetic-field excitation. Under the no-field baseline condition, as shown in Figure 4a–c, the arc column is governed jointly by mechanical separation, electric forces, thermal buoyancy, and local gas disturbances. The arc region therefore exhibits a relatively broad clustered or irregular flame-like morphology, with the yellow luminous region extending over a comparatively large lateral range. Although the arc column is continuously elongated, neither its deflection direction nor arc-root attachment is subject to a stable constraint, resulting in the random drift characteristic of natural arcing. The 0 Hz static transverse field represents the other limiting case, as shown in Figure 4d–f, where the current-carrying channel bends persistently toward one side and exhibits pronounced dragging behavior. Particularly during the middle and late stages of arcing, the outer yellow luminous region expands markedly in the lateral direction, indicating that the sustained unidirectional Lorentz force alters the spatial equilibrium of the plasma channel and can enhance heat exchange between the arc column and the surrounding cooler gas [23].

Between these two limiting cases, a periodically reversing magnetic field suppresses both the irregular accumulation associated with natural arcing and the prolonged unidirectional elongation induced by a static field. At 5 Hz, the nominal half-period is 100 ms, providing sufficient time for arc-column deformation and migration within each half-cycle and thereby allowing relatively large transient deflections to develop, although more pronounced local residence and abrupt jumps may also occur. At 10 Hz, continuous bending, elongation, and contraction remain observable within the 50 ms half-period, while the luminous core maintains good conductive continuity and prolonged one-sided expansion is restricted. When the rotation frequency increases to 30 Hz, the half-period decreases to only 16.7 ms, and the rapid field reversal increasingly competes with the finite deformation response of the plasma channel. Consequently, large-scale deflection within a single forcing interval is weakened, and the arc column appears macroscopically more compact, with only small-amplitude disturbances and occasional deflections of the current-carrying channel. Yamamoto et al. [22] similarly observed a reduction in the oscillation amplitude of thermal plasmas in the high-frequency magnetic-field regime, indicating that increasing frequency does not necessarily strengthen magnetic driving because the response is constrained by plasma thermal inertia and the rate at which attachment states can be renewed.

The morphological evolution described above further reveals the history-dependent response of the current-carrying channel under a periodic magnetic field. After magnetic-field reversal, the arc-column temperature, particle density, and electrode-surface hot spots do not immediately disappear, allowing the subsequent discharge to continue evolving along the previously curved channel or from the residual thermal plume and arc-root hot spots. At 5 Hz, the existing channel has a relatively long time to develop, so competition between the residual and newly driven channels becomes more pronounced after field reversal, favoring large-scale elongation and abrupt contraction. At 10 Hz, the shorter unidirectional forcing interval allows the residual channel to maintain conductive continuity without persistently locking the arc root to one region. At 30 Hz, the thermal-state difference between adjacent half-cycles is further reduced, and the alternating lateral impulses acting on the arc column tend to cancel at the macroscopic scale. Qian et al. [11] reported that the coupled interaction between the arc column and arc root must accumulate to a sufficient thermal or force imbalance before an actual arc-root detachment event occurs. Therefore, the progressive reduction in the visible oscillation amplitude in Figure 4 can be interpreted as a transition from macroscopic arc-column deflection to rapid disturbances that do not fully develop into spatial reconfiguration under the externally applied magnetic force.

### 3.2. Arc-Root Dynamic Response

The macroscopic deflection of the arc column is accommodated through arc-root attachment, migration, and reattachment on the contact surfaces. To further quantify differences in arc-root motion under different magnetic-field conditions, arc-root positions were extracted from the high-speed images using sequential frames sampled at a fixed interval. Before arc-root localization, all images were calibrated using a consistent baseline and spatial scale, and arc-root positions were sampled every 10 frames, corresponding to an 8 ms interval at 1250 fps. Following the coordinate-localization method reported in Ref. [17], the pre-arcing contact point was defined as the origin, while the image coordinate directions were determined from the copper-electrode axis and the normal direction of the carbon-electrode boundary. A narrow high-intensity band near the cathode boundary was selected, and the position of peak intensity was used as an optical proxy for the cathode arc-root location, from which the projected displacement was calculated.

Four dynamic metrics were introduced to characterize arc-root motion. The mean absolute displacement was used to quantify the intensity of arc-root migration and was defined as:

(6) $$D_{r}=\frac{1}{N}\sum_{i=1}^{N}\left|s_{i}\right|,$$

where *s_i_* denotes the projected displacement at the *i*th sampled point and *N* is the number of sampled points in the trajectory. The displacement standard deviation was used to characterize the dispersion of the arc-root trajectory and was expressed as:

(7) $$\sigma_{r}=\sqrt{\frac{1}{N-1}\sum_{i=1}^{N}{\left(s_{i}-\bar{s}\right)}^{2}},$$

where $\bar{s}$ denotes the mean displacement of the trajectory. The drift width was used to quantify the arc-root sweep range within a single trajectory and represents the maximum span of the projected displacement, defined as:

*W_r_* = max(*s_i_*) − min(*s_i_*). (8)

**Figure 5:**
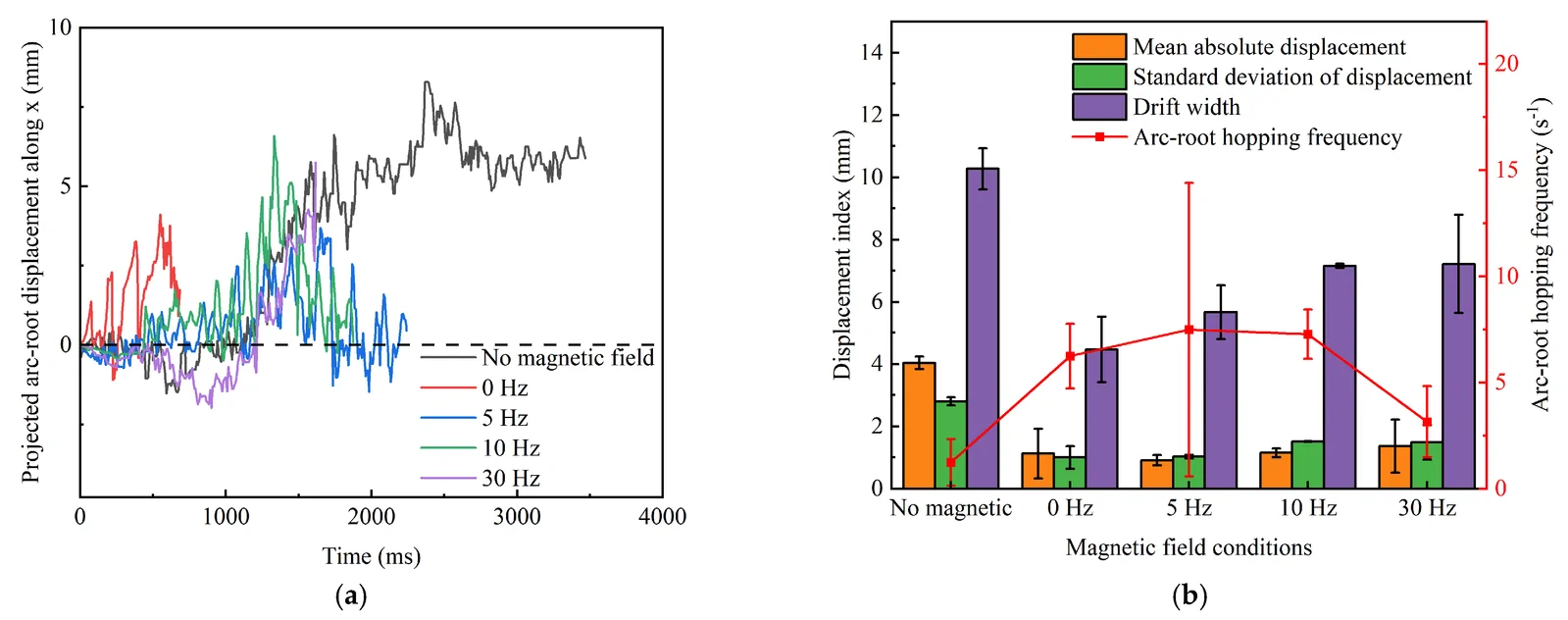
Arc-root dynamics under different magnetic field conditions. (**a**) Representative time histories of cathode arc-root displacement projected along the *x*-axis defined in Section 2.3; (**b**) mean absolute displacement (*D_r_*), displacement standard deviation (*σ_r_*), drift width (*W_r_*), and hopping frequency (*f_j_*) of the arc root.

Meanwhile, the absolute displacement increment between two adjacent sampled points was used to identify discrete arc-root jump events, expressed as:

Δ*s_r_* = |*s_i_* − *s_i_*_−1_|, (9)

when Δ*s_r_* ≥ 1 mm, the displacement was counted as one discrete jump event. The jump frequency, *f_j_*, was then calculated by dividing the total number of jump events, *N_j_*, by the corresponding arcing duration, thereby characterizing the discrete renewal rate of arc-root attachment positions per unit time. Owing to the 8 ms sampling interval, rapid displacement events occurring between sampled frames may be missed or merged. Therefore, the calculated jump frequency represents the observed event rate at the adopted temporal resolution. Figure 5a shows representative time-dependent arc-root drift trajectories under the different magnetic-field conditions, while Figure 5b and Table 3 present the mean values of the dynamic metrics obtained from three independent repeated tests, with the error terms representing the sample standard deviation among the repeated tests.

Combined analysis of the trajectories in Figure 5 and the statistical parameters in Table 3 shows that, under the no-field condition, all three repeated tests exhibited pronounced long-term unidirectional drift of the arc root, consistent with the arc morphologies observed in Figure 4a–c. As arcing proceeded, the arc root migrated slowly and continuously in the positive direction, producing a cumulative displacement of approximately 6–10 mm. The trajectories were dominated by gradual and continuous drift, with only a few abrupt local changes in position. The mean absolute displacement *D_r_* reached 4.038 mm and the displacement standard deviation *σ_r_* reached 2.799 mm, both markedly higher than those under the applied magnetic-field conditions. The no-field case also exhibited a longer arcing duration because, in the absence of external magnetic constraint, the arc root could undergo prolonged random migration and spontaneous reattachment. This unconstrained cumulative drift therefore produced a large spatial excursion, with *W_r_* reaching 10.271 mm, the highest among the five conditions.

Under the 0 Hz static field, repeated episodes of directional migration followed by abrupt reverse jumps were observed. Similar pronounced directional arc-root migration under an applied magnetic field has also been reported in pantograph–catenary arc experiments [24]. During the first approximately 0–75 ms of arcing, the arc root migrated continuously from its initial position to about +1.37 mm and then rapidly returned to the vicinity of the initial position within less than 10 ms. Between 184 and 217 ms, it migrated again from approximately +1.12 to +2.29 mm, followed by an abrupt jump to about −1.10 mm during 217–225 ms, corresponding to a single-event Δ*s_r_* exceeding 3 mm. The arc root then resumed its positive migration from approximately 259 ms and reached about +3.27 mm before rapidly returning again to the vicinity of the initial position at around 400 ms. After reattachment, positive migration resumed and the maximum displacement reached approximately +4.11 mm at around 550 ms. This recurring trajectory pattern demonstrates that, under a static transverse magnetic field, arc-root motion is characterized by directional forcing, detachment, and subsequent reattachment.

When the magnetic field was changed from static to periodically rotating, the arc-root motion shifted from unidirectionally biased forcing to reciprocating migration with alternating positive and negative displacements. The 5, 10, and 30 Hz trajectories in Figure 5a crossed the zero-displacement position repeatedly, indicating that periodic field reversal suppressed the long-term accumulation of unidirectional arc-root drift. Across the repeated tests, the 5 Hz condition exhibited an observed *f_j_* of 7.49 ± 6.91 s^−1^, indicating substantial inter-test variability. This indicates that the low-frequency rotating field had already begun to modify the arc-root attachment state, while the subsequent reattachment and migration process remained sensitive to the initial attachment position and local surface condition, resulting in pronounced test-to-test variability. At 10 Hz, the arc-root trajectories exhibited more concentrated statistical characteristics. Although *W_r_* increased to approximately 7.16 mm, its standard deviation across the three repeated tests was only about 0.065 mm, while *σ_r_* was approximately 1.51 mm, indicating a marked reduction in overall variability. Thus, although this frequency did not minimize the arc-root excursion, it exhibited smaller observed inter-test variability in drift width than 5 Hz, suggesting more reproducible spatial modulation under the tested conditions.

When the frequency was further increased to 30 Hz, *W_r_* remained approximately 7.22 mm, close to that at 10 Hz, but its inter-test standard deviation increased to about 1.58 mm. Both *D_r_* and *σ_r_* likewise exhibited greater variability among the repeated tests. Meanwhile, the observed *f_j_* decreased to approximately 3.15 s^−1^, less than half that at 10 Hz. This decrease may reflect both changes in arc-root attachment dynamics and unresolved rapid events at the adopted sampling interval. Together with the increased variability in drift width, these findings suggest less reproducible arc-root motion at 30 Hz, consistent with a possible mismatch between magnetic-field reversal and arc response.

### 3.3. Electrical Conduction Characteristics and Energy Response

**Figure 6:**
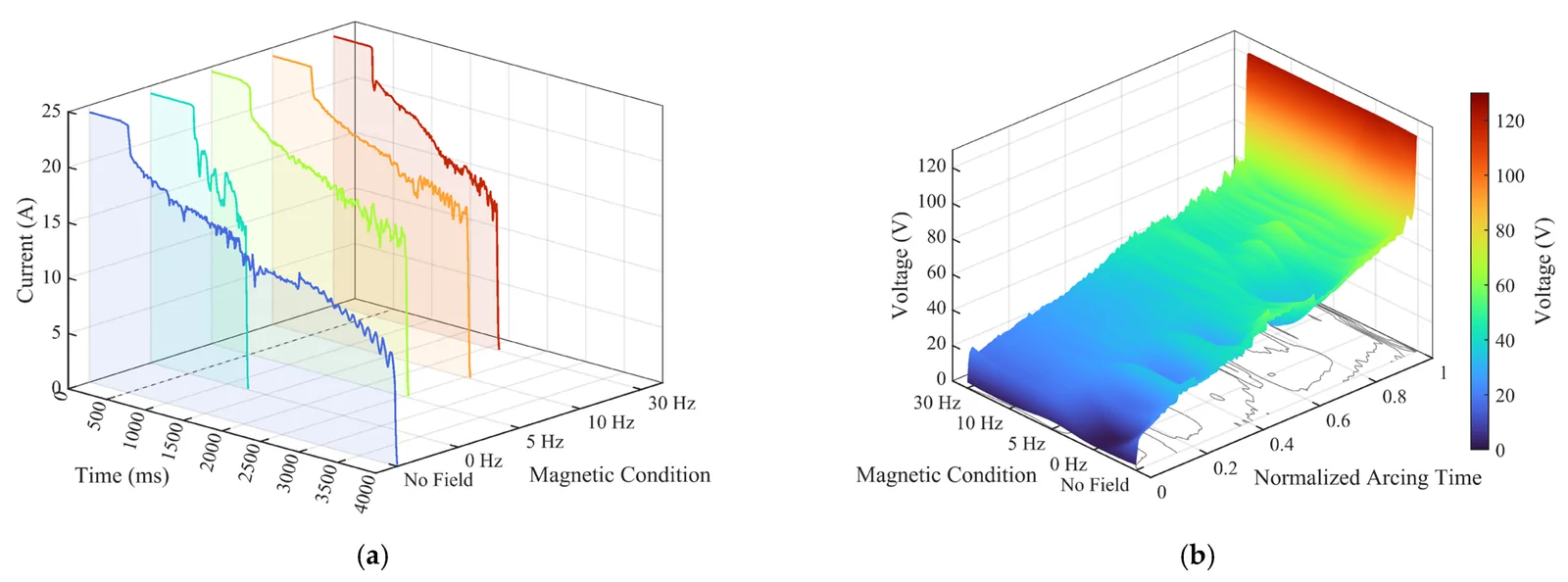
Time-domain electrical response under different magnetic-field conditions. (**a**) Arc-current histories of the first repeated test; (**b**) arc-voltage evolution versus normalized arcing time.

**Figure A1:**
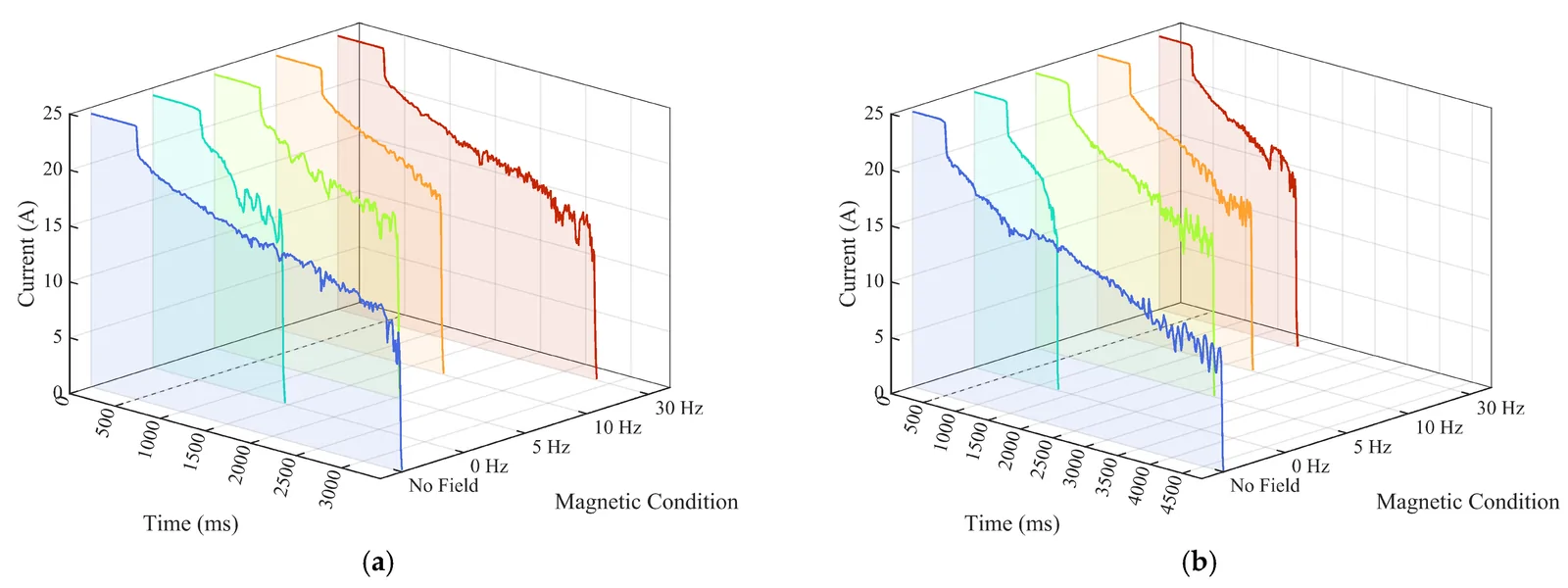
Supplementary repeated-test results of arc-current evolution under different magnetic-field conditions. (**a**) Repeated Test I; (**b**) repeated Test II.

The voltage and current during arcing reflect the ability of the discharge channel to sustain electrical conduction. For a given power supply and series load, an increase in arc voltage is generally accompanied by a decrease in circuit current. Figure 6 compares the electrical responses under different magnetic-field conditions in the time domain. Figure 6a presents the time-varying arc-current curves from the first repeated test for each condition on a common time base, including the 500 ms steady-current conduction period before arc initiation. This pre-arcing segment is shown for reference and is not included in the arcing-period calculations. Figure 6b normalizes the arcing duration of each condition to a range of 0–1 and shows the evolution of arc voltage with arcing progress under different magnetic-field conditions, thereby reducing the influence of differences in absolute arcing duration on stage-by-stage comparisons. The current traces from the remaining two repeated tests are provided in Figure A1 of Appendix A.

As shown in Figure 6a, the current in all five conditions rapidly decreased from the steady-state level to approximately 21 A, while the differences induced by the magnetic field became evident mainly during the sustained arcing stage. Under the no-field condition, after the initial rapid decrease following arc initiation, the current entered a prolonged stage of gradual decay. In the representative waveform, the current remained at approximately 15–18 A during 1–2 s and then gradually decreased as the separation distance increased. Two transient restrike events occurred during 2–3 s, followed by a pronounced low-current tail in the late arcing stage, with the current not falling rapidly to zero until approximately 4 s. This behavior indicates that, without an external magnetic field, the discharge channel can maintain electrical continuity for an extended period through continuous morphological adjustment and arc-root migration, providing the time-domain basis for a relatively large cumulative energy input.

After the transverse magnetic field was applied, arc-column deflection increased the effective arc length, while arc-root detachment and reattachment introduced transient changes in electrical resistance. Consequently, the low-current tail in the late arcing stage was shortened and accompanied by repeated current drops and recoveries. This effect was most pronounced under the 0 Hz condition, where sustained unidirectional forcing caused rapid elongation and earlier extinction. For the three rotating-field conditions, the current waveforms remained similar during the early arcing stage but diverged during the middle and late stages. At 5 and 10 Hz, relatively high current levels were maintained over a longer common arcing interval, together with moderate local fluctuations, whereas the 30 Hz condition entered the low-current regime earlier. These results show that, with the magnetic-field amplitude held constant, increasing the permanent-magnet rotation speed did not continuously improve the stability of current conduction, indicating a distinct frequency-dependent response to the periodically reversing magnetic field.

Examination of the local waveform structure further shows that current disturbances and arc-root events share a common time-domain origin. During continuous arc-root sliding along the electrode surface, the arc length and channel cross-section vary gradually, and the current therefore exhibits predominantly progressive decay. When an existing attachment point suddenly detaches and a new conductive channel is established at another location, the effective arc length, near-electrode voltage drop, and local conductivity change simultaneously, producing abrupt current drops, recoveries, or short-duration spikes. The low-current tail observed in the late stage of the no-field condition is mainly associated with slow arc-root drift and repeated weak restrikes, whereas the frequent current drops and recoveries at 0 Hz are consistent with the repeated directional migration and rapid return observed in Figure 5. This correspondence indicates that arc-root reattachment can induce voltage fluctuations and that changes in attachment position can also perturb the electrical signals, consistent with the three-dimensional transient thermal-plasma torch simulations reported by Perambadur et al. [25]. Among the rotating-field cases, the longer half-period at 5 Hz allows each channel-deformation process to develop more fully, making local abrupt events more sensitive to the initial state, whereas 10 Hz distributes these events over a more stable temporal scale. At 30 Hz, the current entered the low-current regime earlier, suggesting that rapid field reversal did not maintain conductive-channel continuity as effectively as at 10 Hz. A possible mismatch between magnetic-field reversal and the arc response may contribute to this behavior. Figure 6b further shows that the arc voltage generally increases with normalized arcing progress under all conditions, with superimposed local fluctuations of different amplitudes corresponding to the current decay observed in Figure 6a. The normalized time axis is used only to compare stage-dependent evolution among the different conditions and does not represent their absolute arcing durations.

The arcing duration *T_a_* and cumulative arc energy *E_a_* were used to characterize the arc-discharge behavior. The arc-initiation time *t_s_* was identified from the transition from stable contact conduction to arc discharge during electrode separation, using the synchronized voltage and current signals. The final extinction time *t_e_* was defined as the time at which the discharge current decreased to zero without subsequent recovery. Temporary interruptions followed by restrikes were therefore included within the same arcing event. The arcing duration *T_a_* was then defined as the absolute time interval between *t_s_* and *t_e_*. The cumulative arc energy *E_a_* characterizes the thermal energy input to the contact pair during arcing. Because the measured voltage includes the voltage drops associated with the electrodes and circuit resistance, the corresponding Joule loss was evaluated to reduce the resulting error in the energy calculation. Before the experiments, the equivalent resistance was measured three times at 23 °C, and its mean value, denoted as *R_e_*, was approximately 2.69% of the prescribed load resistance. *R_e_* was therefore treated as a constant correction term and subtracted from the energy calculation, while dynamic resistance variations caused by temperature rise were not considered. The corrected *E_a_* can thus be expressed as:

(10) $$E_{a}=\int_{t_{s}}^{t_{e}}U\left(t\right)I\left(t\right)\;dt-\int_{t_{s}}^{t_{e}}I^{2}\left(t\right)R_{e}\;dt,$$

where the integration interval covers the entire arcing duration, and *U* and *I* denote the synchronously measured interelectrode voltage and circuit current, respectively. The integration time is expressed in seconds (s), and the resulting arc energy is reported in joules (J).

**Figure 7:**
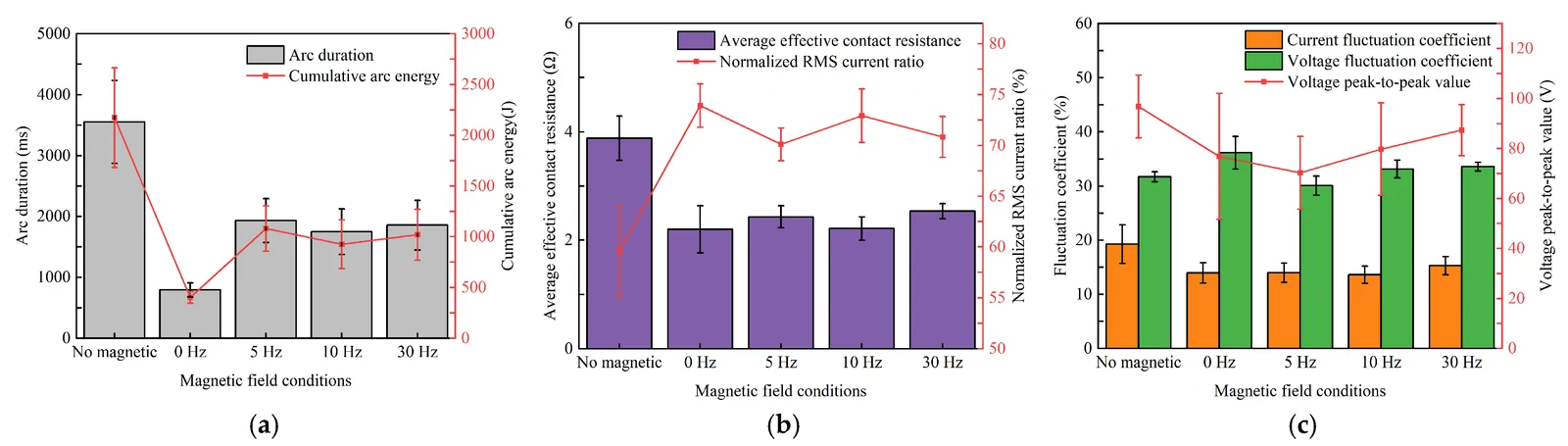
Arc-discharge and current-collection characteristics under different magnetic-field conditions. (**a**) *T_a_* and *E_a_*; (**b**) *R_a_* and *γ_I_*; (**c**) *δ_I_*, *δ_U_* and ∆*U_pp_*.

In addition, five electrical indicators were introduced to further evaluate the dynamic stability over the entire arcing process, namely the mean effective arc-channel equivalent resistance *R_a_*, normalized RMS arcing-current ratio *γ_I_*, current fluctuation coefficient *δ_I_*, voltage fluctuation coefficient *δ_U_*, and voltage peak-to-peak value ∆*U_pp_*. Among these indicators, *R_a_* characterizes the electrical resistance of the arc channel under different operating conditions, with a larger value indicating stronger resistive behavior. To quantify the intrinsic resistive characteristics of the arc-discharge channel while excluding the contribution of the inherent system resistance, the corrected *R_a_* is defined as:

(11) $$R_{a}=\frac{1}{N}\sum_{i=1}^{N}(\frac{U_{i}}{I_{i}}-R_{e}),$$

where *U_i_* and *I_i_* denote the instantaneous circuit voltage and current at the *i*th sampling point, respectively, and *N* is the total number of sampling points within the arcing interval. The parameter *γ_I_* is defined as the ratio of the root-mean-square current during the arcing interval to the reference current and is expressed as:

(12) $$\gamma_{I}=\frac{I_{\mathrm{rms}}}{I_{0}}\times 100\%,$$

where *I_rms_* is the root-mean-square value of the arcing current, reflecting the combined effects of current magnitude and fluctuation on energy transfer, and *I*_0_ is the reference current measured after 30 s of steady-state conduction and taken as the mean of three repeated measurements. This ratio does not represent the overall current-collection or energy-transfer efficiency. The dimensionless coefficient *δ_I_* characterizes the relative current fluctuation [26], with a smaller value indicating greater current stability and a larger value indicating more pronounced variation in the conductive state. It is expressed as:

(13) $$\delta_{I}=\frac{\sqrt{\frac{1}{n}\sum_{i=1}^{n}{\left(I_{i}-\bar{I}\right)}^{2}}}{\bar{I}}\times 100\%,$$

where $\bar{I}$ is the mean current over the arcing interval. The *δ_I_* reflects, to some extent, the degree of arc-channel reconfiguration throughout the arcing process and is expressed as:

(14) $$\delta_{U}=\frac{\sqrt{\frac{1}{n}\sum_{i=1}^{n}{\left(U_{i}-\bar{U}\right)}^{2}}}{\bar{U}}\times 100\%,$$

where $\bar{U}$ is the mean voltage over the arcing interval. The ∆*U_pp_* captures the maximum voltage excursion during a single arcing event and is particularly sensitive to large transient fluctuations, thereby reflecting extreme events such as arc-root jumps and instantaneous disruption of the conductive channel. It is expressed as:

∆*U_pp_* = *U*_max_ − *U*_min_, (15)

where *U*_max_ and *U*_min_ are the maximum and minimum voltages within the arcing interval, respectively. Figure 7 presents the arc-discharge and current-collection performance indicators under different magnetic-field conditions. Table 4 summarizes the mean values and standard deviations obtained from three repeated tests.

As shown in Figure 7a, under the no-field condition, *T_a_* and *E_a_* were 3.553 ± 0.679 s and 2175 ± 491 J, respectively. Both parameters decreased markedly after the transverse magnetic field was applied, reaching 0.793 ± 0.117 s and 401 ± 57 J at 0 Hz. The three rotating-field conditions exhibited a similar non-monotonic frequency response in both *T_a_* and *E_a_*, with both parameters improving from 5 to 10 Hz and then increasing again at 30 Hz. These results indicate that magnetic-field application can shorten the arcing process, whereas the energy-reduction effect of the periodic field does not increase monotonically with frequency.

Figure 7b shows that the application of the transverse magnetic field generally reduced *R_a_* while increasing *γ_I_*, revealing a clear correspondence between the two parameters. Combined with Figure 6a, the prolonged low-current arcing stage under the no-field condition increased the proportion of high-resistance states over the complete arcing interval, thereby weakening the effective current-transfer capability during natural arcing. After the transverse magnetic field was applied, *R_a_* decreased to the range of 2–3 Ω, while *γ_I_* increased to approximately 70% or higher. At 0 Hz, *R_a_* decreased to 2.202 ± 0.438 Ω, while *γ_I_* increased to 73.919 ± 2.135%. Among the three rotating-field conditions, *R_a_* and *γ_I_* exhibited a clear frequency dependence. At 5 Hz, *R_a_* and *γ_I_* were 2.434 ± 0.205 Ω and 70.111 ± 1.613%, respectively. At 10 Hz, *R_a_* decreased to 2.215 ± 0.218 Ω, while *γ_I_* increased to 72.923 ± 2.636%. When the frequency was further increased to 30 Hz, *R_a_* rose to 2.539 ± 0.139 Ω, while *γ_I_* decreased to 70.845 ± 2.020%. This trend is consistent with the non-monotonic variations in *T_a_* and *E_a_* shown in Figure 7a, indicating that the 10 Hz condition not only shortened the arcing process but also maintained a relatively favorable conductive state during sustained arcing.

Further comparison between the 0 and 10 Hz conditions shows that their overall *δ_I_* levels were very similar. As shown in Figure 7c, *δ_I_* was 13.935% at 0 Hz and 13.509% at 10 Hz, with only a limited difference in the mean values, although the 10 Hz condition exhibited a lower mean and smaller inter-test scatter. In contrast, *γ_I_* was slightly higher at 0 Hz than at 10 Hz. A similar distinction was observed in the voltage response. The ∆*U_pp_*value at 10 Hz was 79.76 V, slightly higher than the 76.88 V obtained at 0 Hz. In contrast, *δ_U_* showed the opposite trend, reaching 36.168% at 0 Hz but decreasing to 33.153% at 10 Hz. This difference indicates that, compared with the 0 Hz condition, the 10 Hz case could exhibit relatively large instantaneous arc-voltage excursions while maintaining a lower level of relative voltage fluctuation over the complete arcing interval. Taken together, these results indicate a trade-off between the relative RMS current level and fluctuation characteristics, rather than a consistent electrical advantage for either condition.

### 3.4. Macro-/Microscale Characterization and Compositional Evolution of Carbon-Electrode Erosion

Differences in arc dynamics and electrical conduction are ultimately accumulated as thermal damage on the carbon-strip surface. In this study, the post-arcing surface morphologies of the specimens under different conditions were examined at a consistent macroscopic scale, and image-processing techniques were further employed to quantitatively characterize the material-erosion behavior under different magnetic-field conditions. The erosion images were uniformly processed using MATLAB R2024b. Spatial-scale calibration was first performed, and a fixed physical region was selected as the effective analysis area. Candidate damage boundaries were then identified by combining grayscale intensity, local texture, and color-difference features. After manual verification, binary masks were constructed to determine the number of effective erosion pixels *N_e_*, which was converted into the equivalent surface-damage area *A_e_* using the spatial calibration coefficients as:

*A_e_* = *N_e_k_e_*_,*x*_*k_e_*_,*y*_, (16)

where *N_e_* is the total number of pixels with a value of 1 in the binary mask, and *k_e_*_,*x*_ and *k_e_*_,*y*_ are the spatial calibration coefficients in the horizontal and vertical directions of the macroscopic morphology image, respectively.

**Figure 8:**
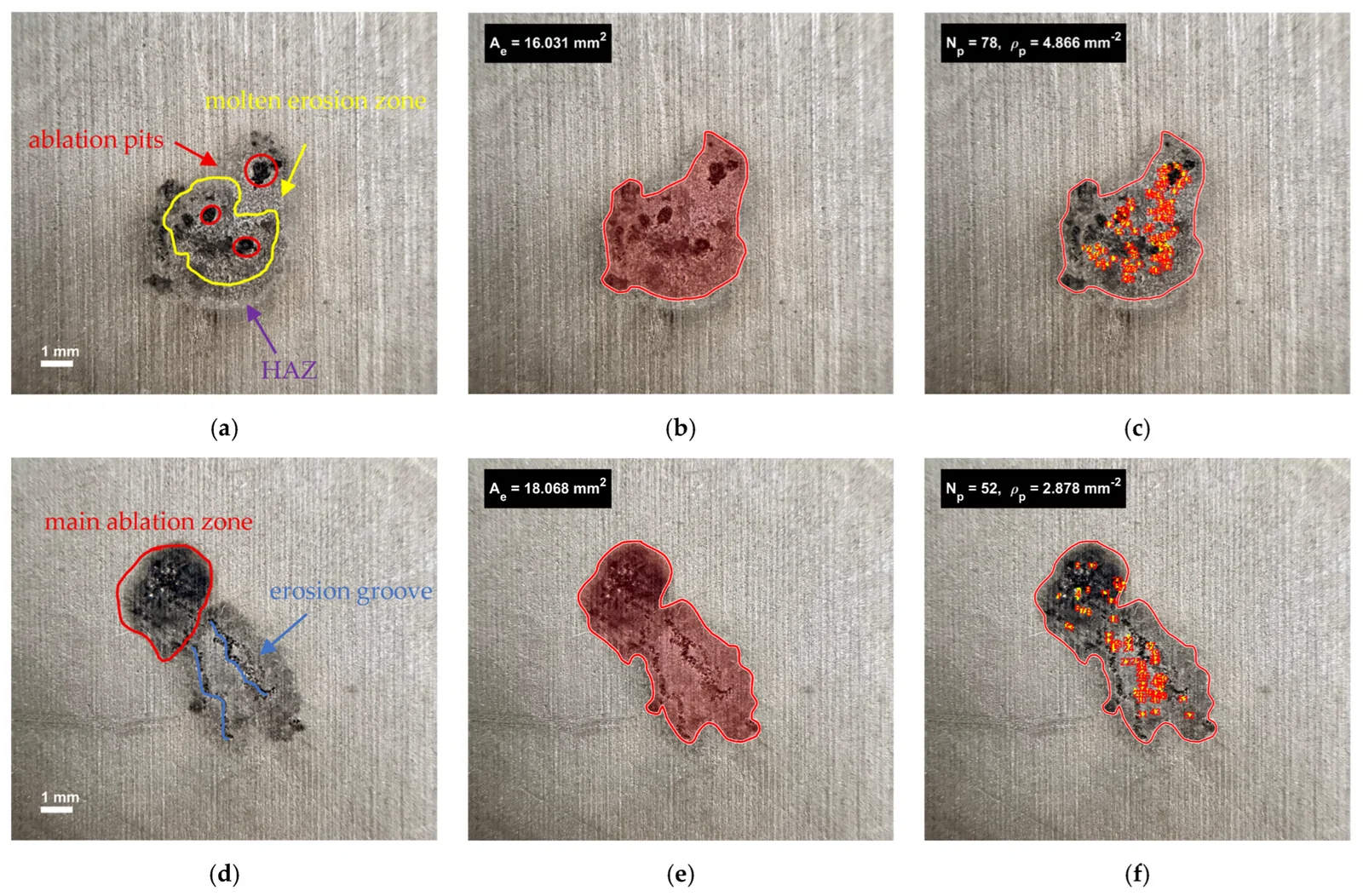

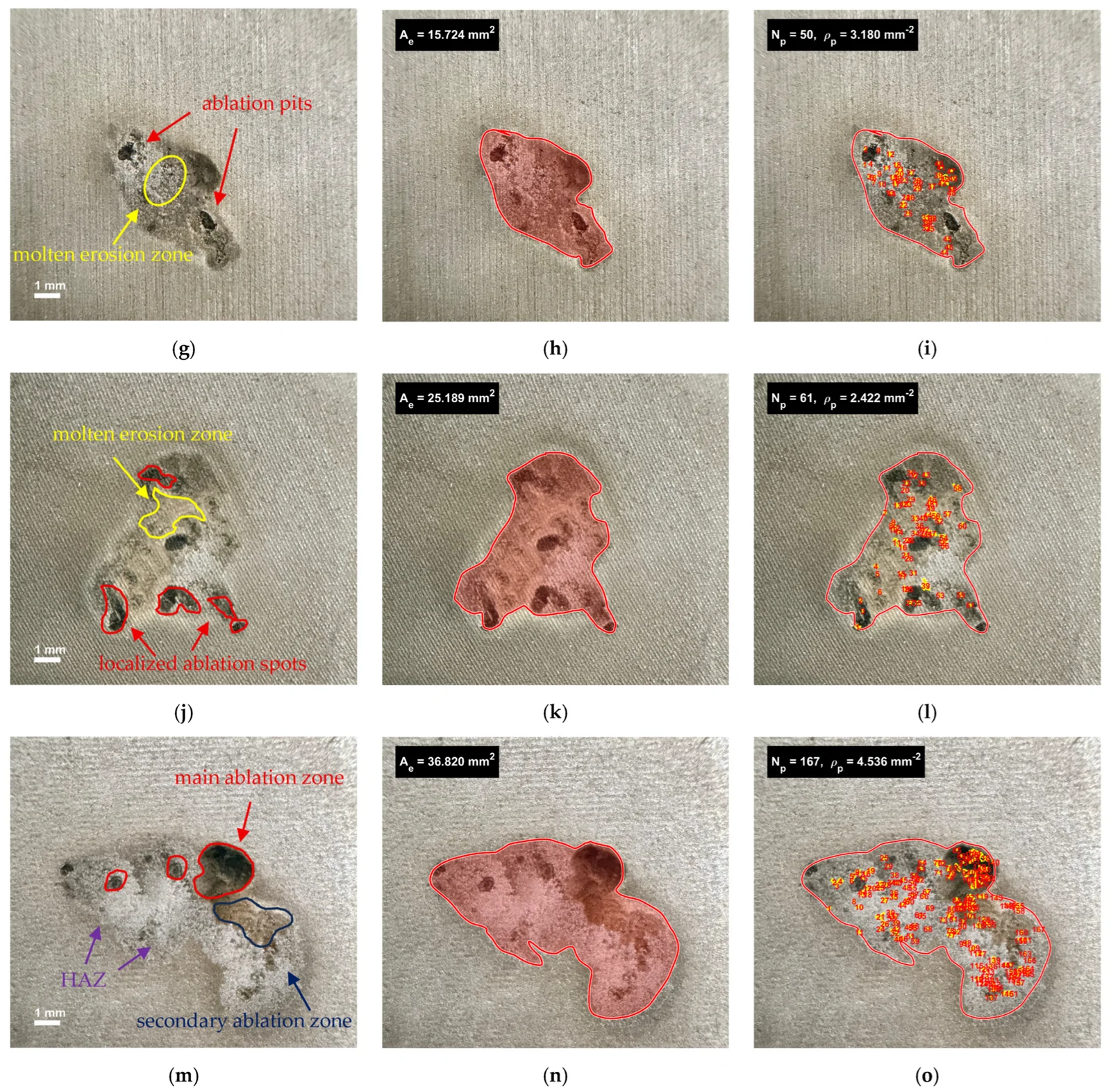
Macroscopic erosion morphologies and quantitative identification results of carbon slide plate under different magnetic-field conditions. (**a**–**c**) The no-field condition; (**d**–**f**) the 0 Hz condition; (**g**–**i**) the 5 Hz condition; (**j**–**l**) the 10 Hz condition; (**m**–**o**) the 30 Hz condition. The shaded regions indicate the identified erosion areas, while the yellow markers and numbers indicate the detected ablation pits and their indices, respectively.

On this basis, local dark pits within the eroded region were enhanced and segmented by thresholding. Isolated noise was removed through connected-component analysis combined with constraints on physical area and shape characteristics, yielding the number of individual ablation pits *N_p_*. The ablation-pit number density *ρ_p_* was subsequently calculated as:

(17) $$\rho_{p}=\frac{N_{p}}{A_{e}},$$

where *ρ_p_* represents the number of discrete ablation pits per unit eroded area and is used to characterize differences among typical damage features such as melting pits, material spallation, erosion grooves, and heat-affected regions. The same image-calibration range and identification parameters were applied to all test conditions to ensure the comparability of the quantitative results. These image-based metrics characterize the two-dimensional extent and spatial distribution of surface damage but do not directly quantify erosion depth, material loss volume or net electrode mass loss. Figure 8 presents the original macroscopic erosion morphologies, identified damage boundaries, and quantitative image-analysis results of the carbon strips under different magnetic-field conditions.

As shown in Figure 8a, under the no-field condition, the eroded region exhibited a large-area irregular clustered distribution, with dark ablation pits of various sizes distributed both inside and around the main erosion zone and surrounded by gray-black heat-affected regions. The local erosion boundaries were relatively dispersed, indicating that the arc-root attachment position changed repeatedly during prolonged free drift and produced multiple centers of intense thermal loading. The longer arcing duration also allowed heat to be repeatedly deposited at randomly distributed locations, resulting in composite damage characterized by a large affected area and a high density of pits, as shown in Figure 8c. After the transverse magnetic field was applied, extensive randomly distributed pitting was suppressed, and the damage pattern began to exhibit spatial characteristics corresponding to the arc-root motion, with this change being particularly evident at 0 Hz. In Figure 8d, in addition to localized ablation cores, the main erosion zone contains elongated damage bands extending continuously along a fixed direction, with adjacent erosion sites locally connected to form complete groove-like structures. This indicates that, although this condition had the shortest arcing duration and the lowest total energy input, sustained unidirectional Lorentz forcing introduced a pronounced directional bias in the thermal-load distribution, producing concentrated damage along a preferred trajectory within a relatively confined region.

After transition to a rotating magnetic field, the directional concentration of the damage was markedly reduced. In Figure 8g,j, both the 5 and 10 Hz specimens exhibit localized dark erosion cores together with interconnected light-gray melt-eroded regions and heat-affected zones, appearing primarily as multiple ablation spots distributed over a finite surface area. At 5 Hz, the overall damage was more compact, with the main erosion zone exhibiting a short band-like distribution. At 10 Hz, the damaged area became somewhat larger, but the local energy-deposition sites were dispersed over a broader surface, producing several interconnected yet spatially more distributed ablation spots. It should be noted that *A_e_* at 10 Hz in Figure 8k was not the minimum among the five conditions. This occurred because the reduction in *E_a_* was accompanied by an expansion of the arc-root activity range, which reduced the concentration of high thermal flux per unit area. The larger *A_e_* therefore reflects spatial dispersion of arc energy rather than an increase in damage severity. When the frequency was increased to 30 Hz, a relatively long continuous erosion band appeared on the surface in Figure 8m, with a large dark primary ablation pit located at one end and multiple secondary pits and lighter heat-affected regions distributed along the direction of motion. All three replicate specimens exhibited large-area, densely distributed pitting with relatively small differences in overall morphology, as shown in Figure 8o.

Combined with the arc-root trajectory analysis, the rapid magnetic-field reversal at 30 Hz did not establish an arc-root migration mode as stable as that observed at 10 Hz. The local conductive channel therefore became more sensitive to the instantaneous attachment state, and localized thermal loading intensified again, indicating that the beneficial effect of low- and intermediate-frequency magnetic-field regulation on macroscopic surface damage weakened as the frequency was further increased. The quantitative statistics of these morphological characteristics are summarized in Table 5.

Overall, the statistical results show that magnetic-field application substantially reduced the relatively high level of material damage observed under the no-field condition. Without an applied magnetic field, *A_e_* reached 33.595 mm^2^, *N_p_* was 162.3, and *ρ_p_* reached 5.106 mm^−2^. Both *A_e_* and *N_p_* also exhibited considerable inter-test scatter, indicating that, during prolonged natural arcing, the final erosion state was strongly dependent on the transient arc-root attachment condition. As shown in Figure 8c, this behavior resulted in multiple sites of concentrated thermal loading distributed over a relatively large and randomly accessed arc-root region. After application of the 0 Hz static transverse magnetic field, all three quantitative parameters decreased to their minimum values, indicating that shortening the arcing duration can substantially reduce the overall thermal load imposed on the carbon specimen. However, Figure 8d shows that the resulting damage developed into continuous groove-like features, suggesting that the discrete-pit-based quantification method has limited sensitivity to directional damage produced under a static magnetic field. In other words, the principal advantage of the 0 Hz condition lies in rapid forced arc extinction and reduced total energy input, whereas sustained arc-root sweeping in a single direction tends to produce concentrated band-like erosion.

Under the rotating-field conditions, *A_e_* was 19.263 mm^2^ at 5 Hz, lower than the 25.828 mm^2^ obtained at 10 Hz. However, the corresponding *ρ_p_* values were 3.266 and 3.114 mm^−2^, respectively, showing similar mean levels with overlapping error ranges. Meanwhile, the standard deviation of *A_e_* at 5 Hz reached 8.540 mm^2^, markedly higher than the 4.737 mm^2^ observed at 10 Hz, indicating greater variability in the erosion extent at 5 Hz. This result is consistent with the large inter-test variability in arc-root jump frequency reported in Table 3 and further indicates that an expanded arc-root migration range does not necessarily imply deterioration of the interfacial discharge state. When the frequency was increased to 30 Hz, the material damage exhibited a clear reversal in trend. *A_e_* increased to 37.966 mm^2^, slightly exceeding that under the no-field condition, while *N_p_* and *ρ_p_* increased to 168.7 and 4.450 mm^−2^, respectively, both substantially higher than those under the other rotating-field conditions. More importantly, all three erosion parameters exhibited relatively small inter-test scatter at 30 Hz, indicating that the elevated erosion level was a reproducible characteristic of this operating condition rather than an anomaly associated with an individual specimen.

Exploratory pairwise comparisons among the three rotating-field conditions showed no statistically significant differences in erosion area, pit number, or pit density after Holm correction (all adjusted *p* ≥ 0.240). Therefore, these differences are interpreted as descriptive trends, with the corresponding arc-root and electrical responses providing additional evidence for their physical interpretation.

These macroscopic damage results exhibit clear cross-scale consistency with the arc-root dynamics. At 30 Hz, the mean arc-root drift width remained close to that at 10 Hz, whereas its inter-test standard deviation increased markedly and *f_j_* decreased from 7.27 to 3.15 s^−1^. The former indicates reduced repeatability of the spatial modulation, while the latter suggests a lower rate of attachment-site renewal and consequently longer local residence of the arc root, which favors concentrated energy deposition [23]. Together, these effects cause thermal-energy deposition at high frequency to exhibit both stronger local concentration and greater randomness in the location of energy input, which accumulate over repeated arcing events into a larger *A_e_* and higher *ρ_p_*. The erosion characteristics observed at 30 Hz therefore provide macroscopic material evidence for the deterioration of magnetic-field regulation in the high-frequency regime.

**Figure 9:**
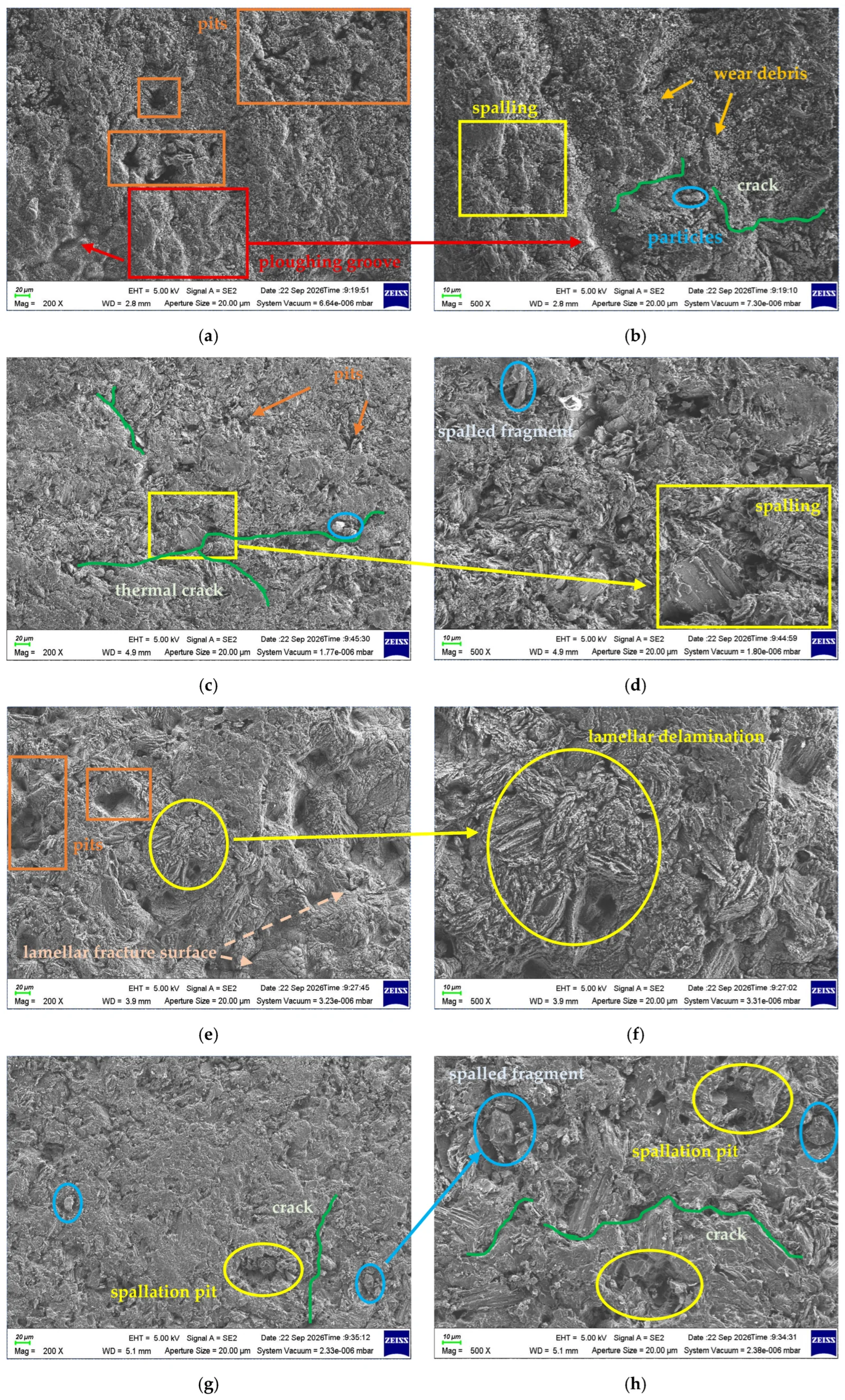

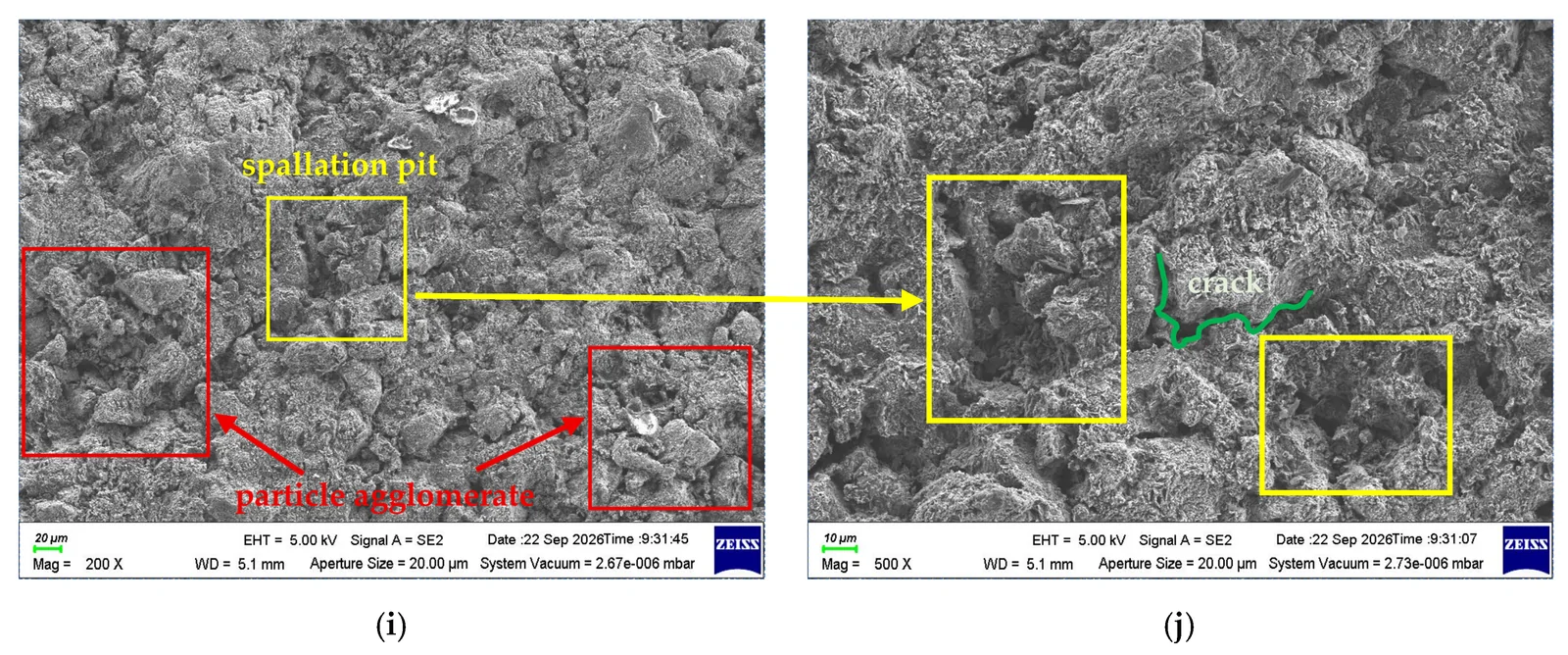
SEM morphologies of arc-eroded carbon-strip surfaces under different magnetic-field conditions at 200× and 500× magnifications. (**a**,**b**) The no-field condition; (**c**,**d**) the 0 Hz condition; (**e**,**f**) the 5 Hz condition; (**g**,**h**) the 10 Hz condition; (**i**,**j**) the 30 Hz condition.

To further identify the local material-removal and surface-reconstruction mechanisms underlying these macroscopic differences, FE-SEM was used to examine representative arc-eroded regions under the five magnetic-field conditions, while EDS was employed to analyze the elemental composition of particulate and flake-like deposits. Figure 9 presents the surface morphologies of the arc-eroded regions on the carbon strips at 200× and 500× magnifications under different magnetic-field conditions. The 200× images primarily reveal the spatial continuity and characteristic scale of the damage features, whereas the 500× images provide more detailed identification of spallation pits, lamellar fracture surfaces, cracks, and particulate deposits.

Under the no-field condition, the specimen surface exhibited pronounced nonuniform topography. Groove-like depressions and irregular spallation pits were visible within the 200× field of view, while the 500× images revealed local open cracks, uplifted lamellar edges, and fine particulate deposits distributed around the pits and across the matrix surface. These features reflect the migration and redeposition of arc-eroded products under arc action. The observed morphology indicates that prolonged natural arcing did not produce uniform thermal loading, but instead repeatedly deposited heat at several randomly distributed attachment sites. The resulting temperature gradients and thermal stresses promoted crack propagation along weakly bonded interfaces within the carbon material, eventually leading to lamellar fragmentation and localized spallation. After application of the transverse magnetic field, pronounced arc-track cracks became visible on the microscopic surface. Enlarged views around these cracks revealed larger angular fragments, opened interlamellar interfaces, and secondary microcracks. Therefore, although the 0 Hz condition produced a smaller macroscopic damaged area, this did not necessarily correspond to a comparable reduction in localized lamellar damage.

At 5 Hz, the directional damage characteristics changed, with relatively large irregular arc-erosion pits and step-like lamellar fracture surfaces becoming more prominent. Nearly parallel lamellar edges and continuous lamellar delamination were also observed in the 500× images. When the frequency increased to 10 Hz, coarse lamellar boundaries and large open pits were reduced, while the overall surface continuity improved. The damage was dominated by dispersed small-scale spallation pits and finer particulate deposits. Combined with the higher arc-root jump frequency and lower inter-test dispersion of the drift width under this condition, these observations indicate that the arc-root thermal input was partitioned into shorter-duration attachment events occurring at continuously changing spatial locations, thereby suppressing sustained crack propagation and the coalescence of adjacent spallation pits. The improvement at 10 Hz was therefore mainly manifested as reduced local thermal concentration and a transition from coarse spallation to finer and more spatially dispersed arc erosion.

At 30 Hz, the microscopic morphology no longer followed this refinement trend. The 200× images showed agglomerated fine particles, arc-erosion pits, and multiscale blocky spallation products, while the 500× images revealed relatively large open spallation pits, lamellar fracture surfaces, and locally uplifted edges, resulting in an overall rougher surface. Combined with the increases in *A_e_* and *ρ_p_* under this condition, the observed erosion morphology is more likely associated with enhanced abrasive damage promoted by repeated thermal cycling. The shorter magnetic-field half-period at 30 Hz may limit the development of arc-column deformation and arc-root relocation within individual half-cycles. This could increase the likelihood of repeated local thermal loading and contribute to the observed coarsening and coalescence of surface-damage features.

These results indicate that the SEM morphologies represent the accumulated surface state resulting from the competition between local thermal cycling and surface coverage processes. During current-carrying sliding, arc migration along the contact interface can shift the location of local energy deposition and generate directional erosion features [27]. Meanwhile, the transient high temperature at the arc root can cause localized melting of the metallic surface, while plasma-jet effects and pressure near the arc root can promote droplet ejection followed by resolidification on adjacent surfaces [28]. Dislocation-mediated plasticity may also contribute to thermomechanical damage on the copper side during mechanical contact and sliding. In molecular dynamics simulations of cold-sprayed Cu–graphene nanoplatelet particles, Wu et al. [29] observed dislocation nucleation and evolution during impact-induced plastic deformation. Although the loading conditions differ from those of the present study, such plasticity may represent a complementary damage mechanism. Therefore, the particulate deposits and rough surface structures observed by SEM may be associated with interfacial material transfer induced by arc heating.

**Figure 10:**
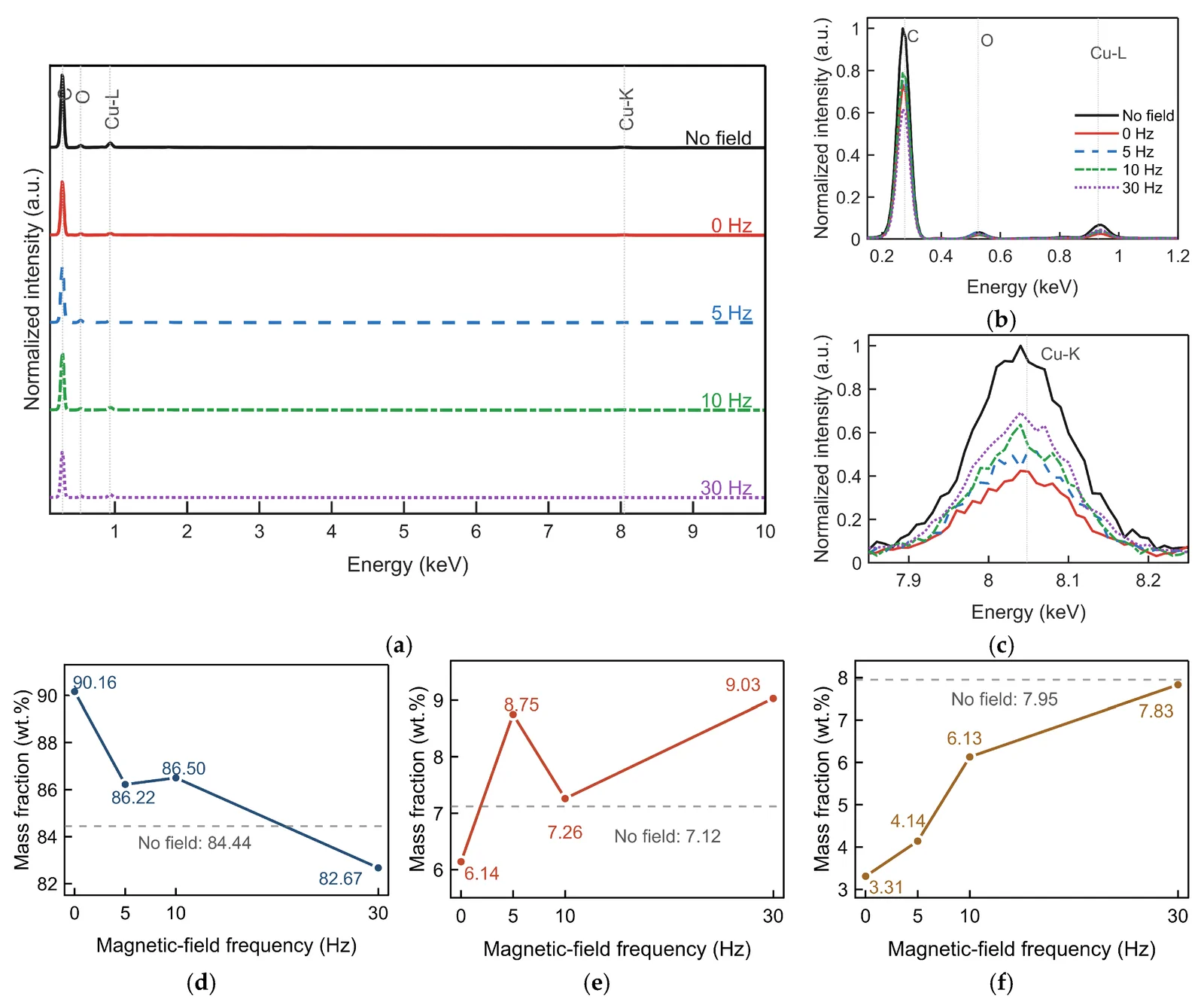
EDS spectra and elemental composition of arc ablation zones under different magnetic field conditions. (**a**) Full EDS spectra; (**b**) enlarged EDS spectra in the low-energy region; (**c**) enlarged EDS spectra in the Cu-K region; (**d**) C mass fraction; (**e**) O mass fraction; (**f**) Cu mass fraction.

To further examine the elemental signatures associated with material transfer and surface reaction, EDS analysis was performed on representative arc-eroded regions under the five magnetic-field conditions. Without an EDS reference for the unexposed carbon surface, the comparison focuses on compositional differences among the post-arcing regions. In particular, the O mass fractions are considered semiquantitative because of their sensitivity to X-ray absorption, matrix effects, and surface roughness. The EDS spectra were dominated by C, O, and Cu, whose combined mass fractions ranged from 99.11% to 99.89%. Trace amounts of Al, Si, Fe, Ca, and S were also detected, but their contents were sufficiently low that they are not discussed further in this study. The EDS results for the arc-eroded regions are presented in Figure 10. The spectral intensities in Figure 10a,b were normalized using the common maximum count among the five datasets, and the spectra in Figure 10a were additionally shifted vertically by equal offsets to avoid overlap. In Figure 10c, the five spectra were normalized using the common maximum count within the local Cu-K energy window to improve the visibility of differences among the weak Cu peaks. The normalized EDS mass fractions characterize the elemental composition of the selected regions rather than the total amount of transferred material. Moreover, the detected O content alone cannot establish specific oxide phases or quantify the extent of oxidation.

Under the no-field condition, the mass fractions of C, O, and Cu were 84.44%, 7.12%, and 7.95%, respectively. The relatively high local Cu content is consistent with the presence of Cu-containing material retained on the carbon-specimen surface after natural arcing. After application of the 0 Hz magnetic field, the C content increased to 90.16%, whereas the Cu content decreased to 3.31%, the lowest among the five conditions, and the O content simultaneously decreased to 6.14%. Considering the shortened arcing duration and the sustained unidirectional magnetic forcing of the arc column under this condition, the static field reduced the cumulative arc-energy input and the overall duration of thermal exposure. This may have limited the opportunity for Cu-containing material to accumulate in the analyzed region, contributing to its lower local Cu mass fraction.

Under the periodic magnetic fields, the local Cu mass fraction increased with increasing frequency. At 5 Hz, the O mass fraction was 8.75%, higher than that under the 0 Hz condition. The relatively long magnetic-field half-period may allow prolonged arc-root residence and repeated local thermal exposure, potentially affecting surface reactions and the retention of oxygen-containing material. When the frequency increased to 10 Hz, the Cu mass fraction rose to 6.13%, while the O mass fraction decreased to 7.26%. Notably, despite the higher local Cu fraction than at 5 Hz, the 10 Hz condition exhibited finer and more dispersed erosion features in Figure 9. This distinction suggests that the severity of local surface damage is not determined solely by the Cu fraction. Instead, the more reproducible arc-root migration observed at 10 Hz may promote the redistribution of thermal loading and interrupt prolonged heating at individual attachment sites, thereby limiting the development of coarse lamellar spallation and continuous erosion features.

When the frequency was further increased to 30 Hz, the local C mass fraction decreased to 82.67%, while the O and Cu mass fractions increased to 9.03% and 7.83%, respectively. Among the five representative regions analyzed, the O fraction was highest at 30 Hz, while the Cu fraction approached that of the no-field condition. At this frequency, the shorter magnetic-field half-period may limit the lateral displacement of the arc column and arc root before the forcing direction reverses. Consequently, nearby surface regions may experience repeated thermal loading rather than effective spatial redistribution of the arc-induced heat input. Such localized thermal cycling could favor the accumulation of Cu-containing residues and oxygen-containing surface material, and contribute to the crack formation, particle agglomeration, and coarse lamellar spallation observed in Figure 9. Together, the compositional differences in Figure 10 and the corresponding SEM features suggest that increasing the frequency to 30 Hz did not sustain the spatial dispersion of thermal loading observed at 10 Hz.

Taken together, the SEM and EDS results suggest that the influence of magnetic-field regulation on erosion cannot be explained solely by changes in local elemental composition. Instead, the observed differences in arc-root motion and electrical response support a mechanism in which magnetic forcing modifies the distribution and duration of local thermal loading, thereby affecting surface deposition and damage evolution. Under the 0 Hz static field, sustained directional forcing shortened the arcing process but produced localized directional damage. At 5 Hz, the relatively long half-period could still favor localized thermal accumulation, whereas the more reproducible arc-root migration at 10 Hz promoted finer and more dispersed erosion features. When the frequency increased to 30 Hz, the shorter reversal interval may limit effective thermal-load redistribution, contributing to renewed surface roughening and coarse spallation. Thus, the observed erosion response reflects the combined effects of arc-root dynamics, thermal loading, and local surface-composition changes rather than a simple monotonic relationship with the Cu or O mass fractions. Although the magnet position and signal-conditioning procedures were kept unchanged, possible contributions from rotation-induced airflow, mechanical vibration, and electrical interference were not independently quantified and therefore cannot be completely excluded.

## 4. Conclusions

Under controlled ambient-air conditions, a rotating permanent magnet was used to generate a periodic transverse magnetic field with an amplitude of 1.0 mT, and the no-field, static-field, and 5, 10, and 30 Hz conditions were systematically compared using synchronized high-speed imaging, electrical measurements, macroscopic damage quantification, and SEM/EDS characterization. The main conclusions are as follows:

1. The static transverse magnetic field continuously bent and elongated the arc column toward one side, whereas periodic field reversal induced alternating deflection and contraction. When the frequency increased from 5 to 10 Hz, the arc retained clearly distinguishable spatial migration, while at 30 Hz the arc tip became more localized and the main arc column exhibited a more compact morphology.
2. The magnetic-field reversal frequency significantly affected the arc-root drift range and jumping behavior, which is consistent with the experimental findings of Cai et al. [30], who showed that variations in alternating magnetic-field frequency can alter the oscillation period and stability of an arc. Increasing the frequency from 5 to 10 Hz enlarged the drift range while improving trajectory repeatability. At 30 Hz, the mean drift width changed only slightly, whereas inter-test variability increased and the observed jump frequency decreased.
3. Application of the magnetic field markedly shortened the baseline arcing duration and reduced cumulative arc energy. In terms of current-collection performance, the 10 Hz condition combined a relatively high normalized RMS current ratio of 72.923% with the lowest current fluctuation coefficient of 13.509%, whereas the 5 Hz condition exhibited the lowest voltage fluctuation coefficient. Yang et al. [31] similarly identified a favorable frequency range for arc stabilization under an alternating transverse magnetic field, although the preferred frequency under their airflow conditions differed from that obtained here, indicating that the appropriate regulation frequency depends on the discharge and environmental conditions.
4. Macroscopic observations of carbon-electrode erosion showed that the applied magnetic field altered the spatial distribution of surface damage through arc-root migration. The no-field condition was dominated by composite damage consisting of densely distributed irregular pits and a large eroded area. Under the static field, the damaged area was substantially reduced, although concentrated grooves developed along a preferred direction. At 5 and 10 Hz, the damage evolved into interconnected but spatially dispersed ablation spots, whereas at 30 Hz the continuous erosion bands expanded and the pit density increased again. These characteristic morphologies are consistent with the carbon-strip arc-erosion features reported by Ding et al. [32,33].
5. The SEM/EDS results revealed frequency-dependent changes in surface-damage morphology and local elemental composition. The representative region under the 0 Hz static field exhibited the lowest Cu mass fraction, while the 10 Hz condition showed finer and more dispersed erosion features, indicating that erosion mitigation was not determined solely by the local Cu content. At 30 Hz, increased local Cu and O fractions were accompanied by renewed surface roughening and coarse spallation, suggesting less effective redistribution of thermal loading. These findings highlight the combined influence of arc-root dynamics, thermal exposure, and surface-composition changes on erosion evolution. The influence of external magnetic fields on interfacial Cu transfer in current-carrying C/Cu contacts was also reported by He et al. [15], providing related evidence from a different contact regime.

Overall, compared with conventional arc-control approaches based on airflow-induced stretching and cooling [34] and emerging surface-modification strategies using graphene coatings for long-term pantograph-strip protection [35], the rotating-permanent-magnet approach proposed in this study regulates arc-root migration and attachment-site renewal through a periodic magnetic field, thereby enabling the current-transfer behavior and spatial distribution of erosion to be adjusted through rotational speed. Under the investigated conditions, this approach provides a comparatively simple and potentially cost-effective means of regulating PCS arcs. Among the tested rotating frequencies, 10 Hz showed a favorable overall response, although a universally optimal frequency was not established. Future work will therefore investigate the coupled effects of magnetic-field amplitude and frequency to identify an appropriate operating range and extend the study to higher-voltage and higher-current DC conditions, as well as AC power-supply conditions. Alongside these extensions, three-dimensional confocal microscopy and weighing the electrodes before and after each test will be considered to complement the present two-dimensional erosion metrics with surface-depth profiles, estimates of material loss volume, and measurements of net electrode mass changes.

## Figures and Tables

**Table 1:** Physical properties of the contact-pair specimens *.

Material Parameters	Cu-CATH-2	Pure Carbon
Hardness	96.2 HV	67 HSD
Specific Heat Capacity (J·kg^−1^·K^−1^)	379.6	709
Density (g·cm^−3^)	8.9	1.82
Electrical Resistivity (µΩ·m)	0.017	12.01
Thermal conductivity (W·m^−1^·K^−1^)	391	—
Bending Strength (MPa)	—	48.1

* The operating temperature of the material is 23 °C. Hardness values are reported on their respective testing scales and are not directly comparable. A dash (—) indicates that reliable material-specific data are unavailable at the stated temperature.

**Table 2:** Magnetic-field test conditions *.

Case	Magnetic-Field Condition	Nominal Transverse Field at Arc-Zone Center	*f_B_* (Hz)	*n* (r/min)	Half-Period (ms)
M1	No applied field	Background	-	-	-
M2	Static transverse field	*B_y_* = +1.0 mT	0	0	-
M3	Rotating periodic field	*B_p_*_,*c*_ = 1.0 mT	5	300	100
M4			10	600	50
M5			30	1800	16.7

* Experimental temperature: 23 °C; relative humidity: 50% RH; total pressure: 101.325 kPa. The listed values are nominal field settings.

**Table 3:** Arc root migration characteristics under different magnetic field conditions (mean ± SD).

Case	*D_r_* (mm)	*σ_r_* (mm)	*W_r_* (mm)	*f_j_* (s^−1^)
M1	4.038 ± 0.198	2.799 ± 0.131	10.271 ± 0.661	1.24 ± 1.10
M2	1.129 ± 0.793	1.002 ± 0.361	4.467 ± 1.053	6.24 ± 1.52
M3	0.916 ± 0.159	1.029 ± 0.059	5.918 ± 1.066	7.49 ± 6.91
M4	1.149 ± 0.139	1.513 ± 0.015	7.155 ± 0.065	7.27 ± 1.16
M5	1.371 ± 0.851	1.489 ± 0.554	7.217 ± 1.580	3.15 ± 1.67

**Table 4:** Arc-discharge and current-collection parameters under different magnetic-field conditions (mean ± SD).

Case	*T_a_* (s)	*E_a_* (J)	*R_a_* (Ω)	*γ_I_* (%)	*δ_I_* (%)	∆*U_pp_* (V)	*δ_U_* (%)
M1	3.553 ± 0.679	2175 ± 491	3.883 ± 0.406	59.570 ± 4.458	19.268 ± 3.557	96.819 ± 12.545	31.712 ± 0.907
M2	0.793 ± 0.117	401 ± 57	2.202 ± 0.438	73.919 ± 2.135	13.935 ± 1.884	76.878 ± 25.273	36.168 ± 2.997
M3	1.933 ± 0.362	1081 ± 224	2.434 ± 0.205	70.111 ± 1.613	13.963 ± 1.770	70.316 ± 14.546	30.094 ± 1.785
M4	1.750 ± 0.376	926 ± 240	2.215 ± 0.218	72.923 ± 2.636	13.509 ± 1.576	79.758 ± 18.485	33.153 ± 1.641
M5	1.857 ± 0.410	1019 ± 252	2.539 ± 0.139	70.845 ± 2.020	15.292 ± 1.688	87.387 ± 10.177	33.599 ± 0.804

**Table 5:** Macroscopic erosion parameters of carbon slide plate under different magnetic fields (mean ± SD).

Case	*A_e_* (mm^2^)	*N_p_* (-)	*ρ_p_* (mm^−2^)
M1	33.595 ± 10.712	162.3 ± 56.5	5.106 ± 0.671
M2	16.920 ± 4.690	45.7 ± 19.3	2.611 ± 0.495
M3	19.263 ± 8.540	66.0 ± 40.4	3.266 ± 0.558
M4	25.828 ± 4.737	84.3 ± 51.2	3.114 ± 1.319
M5	37.966 ± 3.271	168.7 ± 9.6	4.450 ± 0.133

## Data Availability

The original contributions presented in this study are included in the article. Further inquiries can be directed to the corresponding author.

## Abbreviations

The following abbreviations and nomenclature are used in this manuscript:

PCS	Pantograph–catenary system
HV	Vickers hardness
HSD	Shore hardness (D-type scleroscope scale)
MHD	Magnetohydrodynamic
DC	Direct current
AC	Alternating current
RH	Relative humidity, %
FE-SEM	Field-emission scanning electron microscope
EDS	Energy-dispersive X-ray spectroscopy
DAQ	Data acquisition
AFG	Arc fault generator
PET	Polyethylene terephthalate: the chemical formula is (C_10_H_8_O_4_)_n_
fps	Frames per second
px	Pixels
OCL	Overhead contact line
MFC	Mass flow controller
SD	Standard deviation
RMS	Root-mean-square
HAZ	Heat-affected zone
